# A glimpse into the past: phylogenesis and protein domain analysis of the group XIV of C-type lectins in vertebrates

**DOI:** 10.1186/s12864-022-08659-6

**Published:** 2022-06-04

**Authors:** Stefano Barbera, Claudio Cucini

**Affiliations:** 1 Department of Immunology, Genetics and Pathology, The Rudbeck Laboratory, Uppsala University, Uppsala, Sweden; 2 Department of Life Sciences, University of Siena, Siena, Italy

**Keywords:** C-type lectin evolution, CD93, Clec14A, CD248, Thrombomodulin, Whole genome duplications

## Abstract

**Background:**

The group XIV of C-type lectin domain-containing proteins (CTLDcps) is one of the seventeen groups of CTLDcps discovered in mammals and composed by four members: CD93, Clec14A, CD248 and Thrombomodulin, which have shown to be important players in cancer and vascular biology. Although these proteins belong to the same family, their phylogenetic relationship has never been dissected. To resolve their evolution and characterize their protein domain composition we investigated CTLDcp genes in gnathostomes and cyclostomes and, by means of phylogenetic approaches as well as synteny analyses, we inferred an evolutionary scheme that attempts to unravel their evolution in modern vertebrates.

**Results:**

Here, we evidenced the paralogy of the group XIV of CTLDcps in gnathostomes and discovered that a gene loss of *CD248* and *Clec14A* occurred in different vertebrate groups, with *CD248* being lost due to chromosome disruption in birds, while *Clec14A* loss in monotremes and marsupials did not involve chromosome rearrangements. Moreover, employing genome annotations of different lampreys as well as one hagfish species, we investigated the origin and evolution of modern group XIV of CTLDcps. Furthermore, we carefully retrieved and annotated gnathostome CTLDcp domains, pointed out important differences in domain composition between gnathostome classes, and assessed codon substitution rate of each domain by analyzing nonsynonymous (Ka) over synonymous (Ks) substitutions using one representative species *per* gnathostome order.

**Conclusions:**

CTLDcps appeared with the advent of early vertebrates after a whole genome duplication followed by a sporadic tandem duplication. These duplication events gave rise to three CTLDcps in the ancestral vertebrate that underwent further duplications caused by the independent polyploidizations that characterized the evolution of cyclostomes and gnathostomes. Importantly, our analyses of CTLDcps in gnathostomes revealed critical inter-class differences in both extracellular and intracellular domains, which might help the interpretation of experimental results and the understanding of differences between animal models.

**Supplementary Information:**

The online version contains supplementary material available at 10.1186/s12864-022-08659-6.

## Background

The evolution of vertebrate genes has been characterized by impressive chromosome rearrangements that include whole genome duplications (WGDs) as well as chromosomal reorganizations [1, 2]. As suggested by genomic data, showing an expansion of numerous gene families, the transition from cephalochordates to early vertebrates was characterized by multiple polyploidizations by which the ancestral vertebrate has expanded its gene repertoire [3]. Among the gene families that expanded during the evolution of vertebrates, we found of particular interest the group XIV of C-type lectin domain-containing proteins (CTLDcps) and studied their evolution and protein domain composition by collecting publicly available genome annotations of numerous vertebrate species.

The group XIV of C-type lectins is part of the larger superfamily of CTLDcps which consists of seventeen groups in mammals [4]. Four members compose the group XIV of CTLDcps: CD93, Clec14A, CD248, and Thrombomodulin, which have been studied extensively in human and mouse in the contexts of angiogenesis and cancer biology [5–8]. They are type I transmembrane proteins with an extended extracellular domain (ECD) which, from the N-*terminus*, consists of a CTLD, a sushi domain (except for Thrombomodulin) and a variable number of EGF-like repeats: CD93 has five, Clec14A one, CD248 three, and Thrombomodulin six. A Pro/Ser/Thr rich region (known as mucin-like region) extends from the last EGF-like repeat to the transmembrane domain and is responsible for a high degree of glycosylation. Lastly, a short cytotail extends into the cytoplasm [9].

Expression profile analyses have shown peculiar tissue distribution for each of the four proteins. CD93 has been predominantly studied in endothelial cells (EC) although it is also expressed by different cell types such as neurons, monocytes, platelets and hematopoietic stem cells [9]. In ECs it has been shown to regulate adhesion and migration [10–12], and, by binding the extracellular matrix (ECM) protein Multimerin-2 (MMRN2), forms a complex with the β1 integrin inducing fibronectin fibrillogenesis important for EC migration [6, 13]. Moreover, CD93 can be cleaved from the membrane and released in a soluble form (sCD93) which acts as a potent inducer of angiogenesis through its EGF-like tandem repeats [14].

Clec14A expression is considered endothelial specific. Interestingly, the expression of Clec14A is barely detectable in healthy vessels but strongly upregulated in angiogenic tumor-associated blood vessels of non-small cell lung cancer and ovarian cancer tissues [15, 16]. It has been found to regulate EC tube formation and migration in vitro as well as tumor angiogenesis in vivo. Moreover, like CD93 and CD248, Clec14A binds to MMRN2, promoting tumor vascular growth in a Lewis lung carcinoma mouse model [6].

CD248 is the only member of the group XIV that is not expressed by ECs. Instead, it shows elevated expression in fibroblasts, perivascular, mesenchymal and some tumor cells [9]. It was demonstrated that CD248 is able to bind different ECM proteins such as collagen I, fibronectin and MMRN2 [6, 8]. Despite its role in angiogenesis is still controversial, it has been shown that CD248 can act as a regulator of vessel normalization and vascular pruning [9, 17].

Thrombomodulin is expressed in endothelial and lymphatic blood vessels but can also be found in monocytes, neutrophils as well as dendritic cells [18, 19]. Thrombomodulin is the only protein of the group XIV that lacks some key amino acids necessary for the folding of a proper sushi-like domain and the region comprised between the CTLD and first EGF is instead indicated as hydrophobic stretch [9]. Importantly, the absence of a proper sushi-like domain probably accounts for the incapability of Thrombomodulin to bind to MMRN2 [6]. The soluble form of Thrombomodulin, generated after proteolytic cleavage, can modulate angiogenesis by binding the fibroblast growth factor 1 [20, 21]. Besides its role in angiogenesis, Thrombomodulin plays a key role in the coagulation cascade working as an anti-coagulative molecule. Indeed, in this framework, it binds to the serine protease thrombin thus inhibiting the pro-coagulant thrombin-mediated hydrolysis of fibrinogen to fibrin [22].

The human genome annotation shows a peculiar organization of the group XIV of CTLDcps coding genes with total absence of introns in *CD248*, *Clec14A*, and *Thrombomodulin* whereas *CD93* bears a small intron that splits coding and regulatory sequences in two exons. Noteworthy, *CD93* and *Thrombomodulin* lay on the same chromosome in humans, leading to the hypothesis that they may have arisen from a tandem duplication event [9].

Despite protein similarity of the members of the group XIV would suggest a common origin, phylogenetic analyses aimed to understand their phylogenetic relationship as well as their ancestry have never been attempted. Moreover, despite intensive studies on the human and mouse proteins, little is known about the evolution of their protein domains in other vertebrates.

The availability of a multitude of vertebrate genomic, transcriptomic and proteomic data allowed us to retrieve protein orthologs and paralogs used to study the gene synteny of each group XIV member in different vertebrate classes. Importantly, the current genome annotation of *Petromyzon marinus* allows the comparison of syntenic gene families between cyclostome and gnathostome CTLDcps-bearing chromosomes helping reconstruct their evolution across vertebrates. In the present work, gene and protein sequences were collected and used to perform comparative genomics and proteomics analyses. Based on the results of our study, we demonstrated that the group XIV of CTLDcps derived from a common ancestor and inferred an evolutionary scheme of their evolution in vertebrates, showing that modern CTLDcps established independently in cyclostomes and gnathostomes. In addition, we demonstrated the loss of *CD248* in birds as well as the absence of *Clec14A* in monotremes and marsupials. By collecting public data from numerous vertebrate orders, we focused our attention on analyzing extracellular and intracellular domains. We revealed important similarities and differences of the group XIV of CTLDcps that may depend on environmental and species-specific adaptations and point out important differences between vertebrate classes.

## Methods

### Sequence mining and dataset creation

All the vertebrate protein and gene sequences belonging to the group XIV of C-type lectin superfamily and their syntenic genes were identified and retrieved from the Protein (NCBI) database. Doubtful protein annotations as well as taxon-missing sequences were manually checked and recovered *via* pairwise alignment through BLASTp. To retrieve cyclostomes and amphioxi protein sequences from available ill-annotated genomes, gnathostome protein sequences were employed to build HMMER profiles [23]. Due to doubtful annotation, *P. marinus* CTLDcp genes and proteins were named as follows: *CTLDcp*-α (XM_032974273) and *CTLDcp*-β (XM_032974220) genes lying on chromosome 53, *CTLDcp*-γ gene lying on chromosome 17 (XM_032955932), *CTLDcp*-δ gene lying on chromosome 6 (XM_014554877). cDNA sequences of neighbor gene families (*forkhead-box, ovo* and *fos*) lying on the CTLDcp bearing-chromosomes were mined for at least one species *per* vertebrate class and lancelets as described above. A complete list of protein, CDS, cDNA and genome accession numbers used in this study is available in the Additional file 4: Supplementary Table 1.

### Analysis of the group XIV CTLDcps sushi-like domain in *Petromyzon marinus*

In order to compare the sushi-like domain of the group XIV CTLDcps in *P. marinus*, protein domains were predicted using Batch-CD Search NCBI using the CDD database [24]. Since Batch-CD Search failed to predict the sushi-like domain, we manually retrieved the protein region comprised between the CTLD and the first EGF-like (for CTLDcp-α, CTLDcp-β and CTLDcp-δ) or the transmembrane domain (for CTLDcp-γ). To understand if *P. marinus* CTLDcps showed typical amino acids required for the folding of the sushi-like domain [25], we aligned the retrieved sequences using PRALINE toolbox [26–28]. Structural insights of the sushi-like domain folding were obtained by means of the automated protein structure homology-modelling server SWISS-MODEL [29].

### Synteny identification and analysis

Disposition of the syntenic genes was obtained by manually locating each syntenic gene position in the chromosome following the NCBI database annotation. Gene synteny was visualized using Simple Synteny web tool [30]. The chromosome block interval analyzed for synteny was ~ 20Mbp in each direction of the CTLDcp gene of interest, although in many cases this definition encompassed the entire chromosome. One representative species *per* vertebrate class and a minimum of 15 syntenic genes *per* *locus* were used for the analysis.

### Phylogenetic analyses and tree topology tests

For the phylogenetic reconstructions, we utilized well-annotated and largely reported species, including model organisms when possible and employing two representative species *per* vertebrate class. Species employed for the phylogenetic reconstructions: *Homo sapiens* (human), *Mus musculus* (house mouse), *Monodelphis domestica* (gray short-tailed opossum), *Ornithorhynchus anatinus* (platypus), *Gallus gallus* (chicken), *Corvus moneduloides* (new Caledonian crow), *Lacerta agilis* (lizard), *Trachemys scripta elegans* (red-eared slider), *Xenopus tropicalis* (western clawed frog), *Microcaecilia unicolor* (tiny Cayenne caecilian), *Danio rerio* (zebrafish), *Latimeria chalumnae* (coelacanth), *Carcharodon carcharias* (great white shark), *Scyliorhinus canicula* (small-spotted catshark) *Petromyzon marinus* (sea lamprey), *Lethenteron camtschaticum* (arctic lamprey), *Entosphenus tridentatus* (pacific lamprey), *Eptatretus burgeri* (inshore hagfish), *Branchiostoma floridae* (Florida lancelet) and *Branchiostoma belcheri* (Belcher’s lancelet). All phylogenetic analyses were performed using amino-acid or cDNA data matrices automatically aligned using the Aliview built in MUSCLE3.8 aligner [31, 32] and manually revised. Alignments were employed for a Maximum Likelihood (ML) analysis using 10,000 iterations of ultrafast bootstrap in IQ-TREE [33]. Finally, the best evolutionary model was selected during the run with ModelFinder Plus [34]. Rooting was performed on *B. belcheri* and *B. floridae* homologous sequences, which were employed as outgroups. All the final trees were visualized using iTOL [35]. To assess orthology between cyclostome and gnathostome proteins, we constrained ten distinct tree topologies and tested them using five different statistical methods (bp-RELL, p-KH, p-SH, c-ELW, p-AU) through IQ-TREE. Topologies which showed a significant outcome in all the statistical tests were accepted and discussed in the Results section.

### Protein domain identification and Ka/Ks ratio

Protein domains were identified for all the vertebrate taxon including one representative species per order through the Batch-CD Search NCBI using the CDD database [24]. EGF-like domains were manually revised and categorized based on their typical signatures according to the current PROSITE annotation [36]. EGF-like domains containing the CxCx(5)Gx(2)C (PS00022) or CxCx(2)[GP][FYW]x(4,8)C (PS01186) signature were considered EGF 1 or EGF 2, respectively [37].

EGF 2 containing the NxNNC-x(3,14)-C-x(3,7)-CxxBxxxxAxC-x(1,6)-C-x(8,13)-Cx (PS01187), (where ‘N’: negatively charged or polar residue [DEQN]; ‘B’: possibly β-hydroxylated residue [DN]; ‘A’: aromatic amino acid; ‘C’: cysteine involved in disulfide bond; ‘x’: any amino acid), were considered calcium-binding EGFs (cbEGFs) according to the PROSITE annotation [36]. Predicted EGF-like domains falling in neither of the two categories were annotated as EGF-like. Thrombomodulin-like fifth EGF domains (cl07616) were successfully predicted by Batch-CD Search and named Tme5-EGF. For the sushi-like domain containing region identification, we considered the interval between the CTLD and the first EGF-like domain. Similarly, the mucin-like region was considered starting from the first amino acid after the last EGF-like domain and finishing before the transmembrane region. The CDS of each domain used for the analysis is shown in detail in the Additional files 8, 9, 10 and 11: Supplementary files 1–4. Transmembrane domain prediction was carried out by the TMHMM Server, v.2.0 [38, 39]. Phosphorylation of tyrosine residues laying in the cytoplasmic region was inferred by using the NetPhos3.1 server [40]. Only *consensus* with a score > 0.5 were considered reliable and plotted with WebLogo2.8.2 [41, 42].

Protein domain regions were employed to retrieve the corresponding nucleotide sequences through an in-house python3 script to calculate the Ka/Ks ratio. The genetic dataset was aligned as translated amino-acids with MUSCLE3.8 available in the Aliview software [31, 32]. The Ka/Ks ratio was then calculated with the yn00 method available in PAML [43] as previously described [44], and plotted in the R environment v.4.1.1.

### Statistical analysis

Data analyses were performed using the statistical function of R software and the values represent the mean ± SD obtained. Statistical differences among groups were evaluated using the One-way ANOVA followed by Tukey’s multiple-comparison test. All *p*-values reported were two-tailed and *p* < 0.05 was considered statistically significant.

## Results

### Study of the group XIV CTLDcps paralogy in gnathostomes

**Fig. 1:**
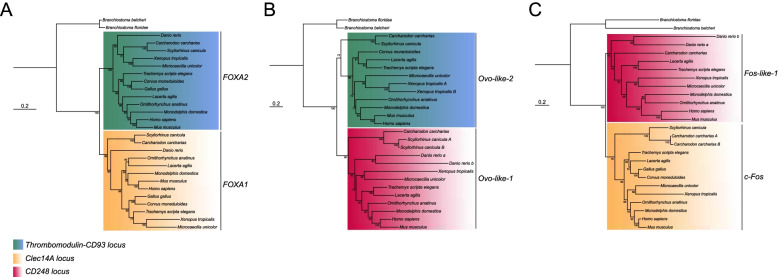
Phylogenesis of highly conserved neighbor gene families of the group XIV of CTLDcps. Phylogenetic reconstruction of *forkhead-box, fos, ovo* gene families of transcription factors. Nucleotide sequences were aligned and subjected to phylogenetic analysis using ML inference. Bootstrap is indicated at each node. Monophyletic subtype clusters, neighbor to a specific CTLDcp, are color-coded and specified in the figure. **A** Phylogenetic analysis of *FOXA2* (*Thrombomodulin-CD93 locus*) and *FOXA1* nucleotide sequences (*Clec14A locus*), ML inference was obtained using the TVMe + R3 evolutionary model. **B** Phylogenetic analysis of *Ovo-like-2* (*Thrombomodulin-CD93 locus*) and *Ovo-like-1* (*CD248 locus*) nucleotide sequences, ML inference was obtained using the SYM + R4 evolutionary model. **C** Phylogenetic analysis of *Fos-like-1* (*CD248 locus*) and *c-FOS* (*Clec14A locus*) nucleotide sequences, ML inference was obtained using the TIM3e + R4 evolutionary model. Capital letters beside the species indicate duplicated genes lying on the same chromosome, while small letters indicate duplicated genes lying on different chromosomes

**Fig. 2:**
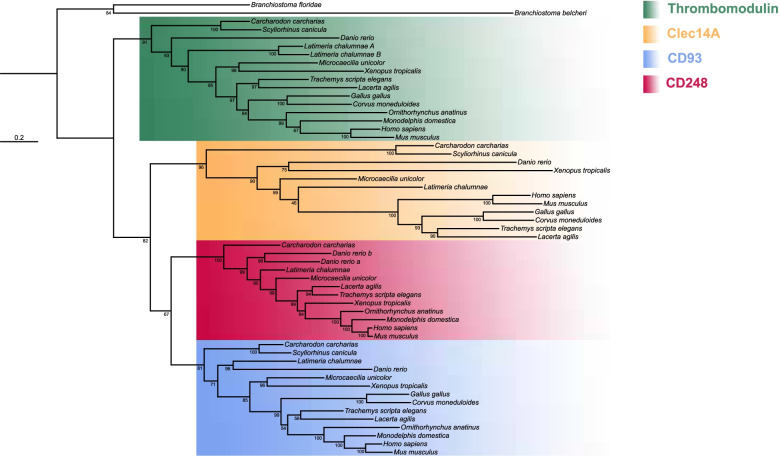
Phylogenetic relationships of gnathostome CTLDcps. Phylogenetic reconstruction of gnathostome CTLDcps amino acid sequences. At each node numbers indicate bootstrap supports of the ML inference analysis. WAG + R4 was the chosen evolutionary model according to Model Finder. Monophyletic subtype clusters are color-coded and specified in the legend. Capital letters beside the species indicate duplicated genes lying on the same chromosome, while small letters indicate duplicated genes lying on different chromosomes

Although CD93, Thrombomodulin, Clec14A, and CD248 are classified in the same protein group, no formal phylogenetic analysis has been conducted to clarify their phylogenetic relationships. Interestingly, it has been previously demonstrated that their bearing chromosomes derived from a series of duplication events as they show typical features of paralogons [45]. To further corroborate these findings, we investigated the evolutionary relationships of three highly conserved transcription factor gene families (*forkhead-box*, *fos* and *ovo*), which we found being neighbors of the CTLDcps genes (Table 1 and Additional file 5: Supplementary Table 2). Our phylogenetic reconstruction showed that these gene families dispose in discrete clusters supporting an ancestral duplication scenario (Fig. 1). Therefore, to unravel the hypothesis that the members of the group XIV share a common ancestry, we retrieved amino acid sequences from the NCBI database and performed a phylogenetic reconstruction of numerous vertebrate species. Using amino acid sequences of CTLD-like proteins of two different lancelet species as outgroups, the resulting ML tree showed the CTLDcps disposition in discrete subtype clusters with a well-supported ancestral node, consistent with the assumption that the group XIV of CTLDcp genes arose from a gene duplication event (Fig. 2). To corroborate these findings, we analyzed the CTLDcp gene *loci* and studied gene synteny of a representative species *per* vertebrate class. A total of 15 gene families were analyzed showing that all the CTLDcps-bearing chromosomes share syntenic gene patterns (Table 1 and Additional file 5: Supplementary Table 2). Interestingly, all the analyzed gnathostome taxa presented *CD93* and *Thrombomodulin* as linked genes since they lie in close proximity on the same chromosome. Moreover, we observed a gene loss of *CD248* and its neighbor genes ascribable to chromosome disruption in birds (Additional file 5: Supplementary Table 2), and the loss of *Clec14A* in monotremes and marsupials, which probably represent a synapomorphy of these groups (Additional file 5: Supplementary Table 2).

**Table 1:**
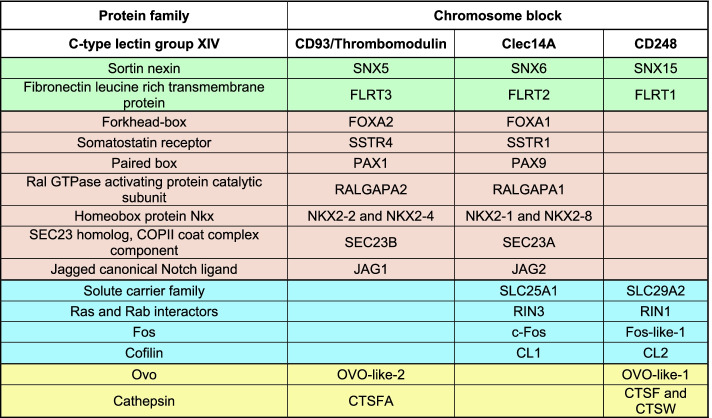
Neighbor gene families of the CTLDcp genes in gnathostomes

Neighboring gene families analyzed for conserved synteny in the *CD93/Thrombomodulin*-, *Clec14A*- and *CD248*-bearing chromosome blocks. Syntenic gene families common to all or two CTLDcps are listed and color-coded. CTLDcps are highlighted in bold. Green lines: syntenic gene families present in all the CTLDcps-bearing chromosomes. Red lines: syntenic gene families present in the *CD93/Thrombomodulin*- and *Clec14A*-bearing chromosomes. Light blue lines: syntenic gene families present in the *Clec14A*- and *CD248*-bearing chromosomes. Yellow lines: syntenic gene families present in the *CD93/Thrombomodulin*- and *CD248*-bearing chromosomes

### Evolution of CTLDcps in cyclostomes and gnathostomes

**Fig. 3:**
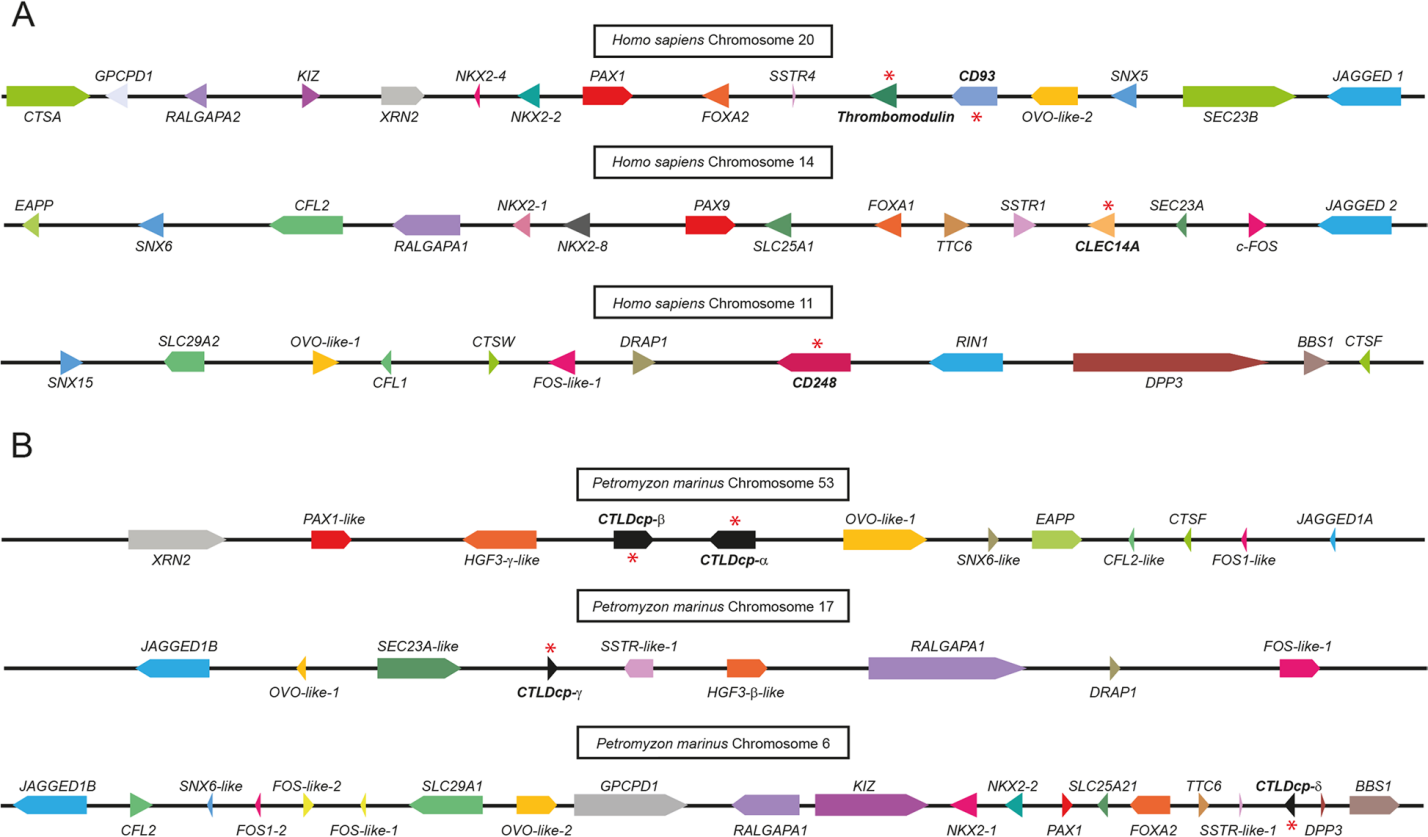
Comparison of *Homo sapiens* and *P. marinus *CTLDcp-bearing chromosomes. **A** Schematic representation of *H. sapiens* CTLDcp *loci* of chromosome 20, 14 and 11. Syntenic CTLDcp gene families share similar colors. CTLDcp names are in bold and a red asterisk marks the gene. **B** Schematic representation of *P. marinus* CTLDcp *loci* of chromosome 53, 17 and 6. Syntenic CTLDcp gene families share similar colors. Lamprey CTLDcps were named CTLDcp-α and –β (chromosome 53), -γ (chromosome 17), and –δ (chromosome 6). CTLDcp names are highlighted in bold and a red asterisk marks the gene

To shed light on the evolutionary history of the CTLDcps, we retrieved CTLDcp genes in *P. marinus*, (namely *CTLDcp*-α, -β, -γ, or -δ), reconstructed their gene *loci*, and compared them to the human CTLDcps-bearing chromosomes (Fig. 3). Importantly, this reconstruction highlighted the presence of numerous shared gene families between the lamprey and the human chromosomes and showed that, on chromosome 53 of *P. marinus*, two CTLDcp genes were found in linkage as *CD93* and *Thrombomodulin* in all gnathostomes (see Fig. 3 and Additional file 6: Supplementary Table 3).

**Fig. 4:**
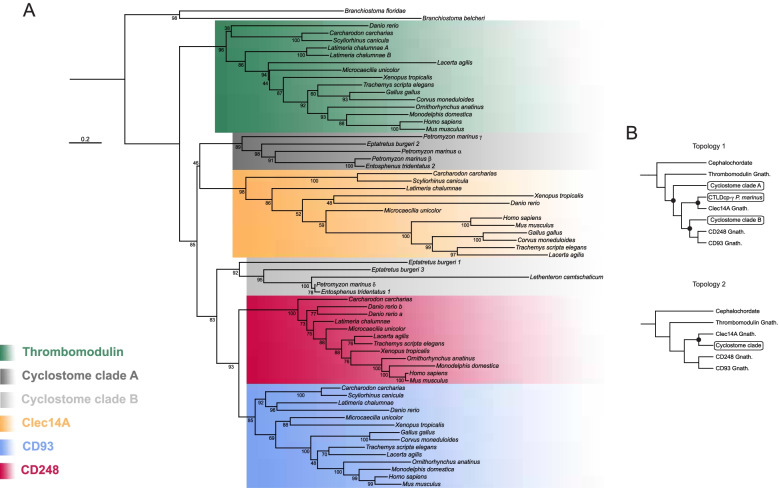
Phylogenetic reconstruction of cyclostome and gnathostome CTLDcps. **A** Tree reconstruction was performed with representative species *per* class using ML statistic approach with WAG + R5 as evolutionary model. Numbers indicate bootstrap values. Protein clusters within the tree are color-coded. **B** Schematic trees representative two statistically significant alternative topologies. The tree on the left shows CTLDcp-γ as Clec14A ortholog, while the tree on the right inferred cyclostomes as monophyletic group and sister to Clec14A. Capital letters beside the species indicate duplicated genes lying on the same chromosome, while small letters indicate duplicated genes lying on different chromosomes

To understand the evolutionary relationships between gnathostome and cyclostome CTLDcp genes, we retrieved additional cyclostome protein sequences from two different lamprey species and one hagfish by building appropriate HMMER profiles for each CTLDcp and performed phylogenetic analyses. In our first reconstruction, none of the cyclostome CTLDcps was unambiguously assigned as gnathostome ortholog and disposed in two distinct clusters (cyclostome clade A and clade B). Indeed, the phylogenetic tree showed sister relationships between cyclostome clade A and Clec14A as well as the cyclostome clade B with the (CD248,CD93) cluster (Fig. 4A). Since hidden paralogy and asymmetric gene retention, that follows gene duplication and loss, can confuse orthology assignment, we tested alternative tree topologies (see Additional file 7: Supplementary Table 4 for details and Additional file 1: Supplementary Fig. 1 for tested tree topologies). Our alternative hypotheses suggested two statistically accepted scenarios of the evolutionary relationships between gnathostome and cyclostome homologs (Fig. 4B). In the topology 1 scenario, CTLDcp-γ of *P. marinus* was found being Clec14A ortholog, while the other cyclostome proteins distributed across two different clades. Similarly, the topology 2 scenario inferred cyclostome proteins as monophyletic and sister to gnathostome Clec14A suggesting that the latter might resemble features of the last CTLDcp common ancestor before the divergence of proto-gnathostomes from proto-cyclostomes.

### *P. marinus* CTLDcp-α lacks the sushi-like domain

**Fig. 5:**
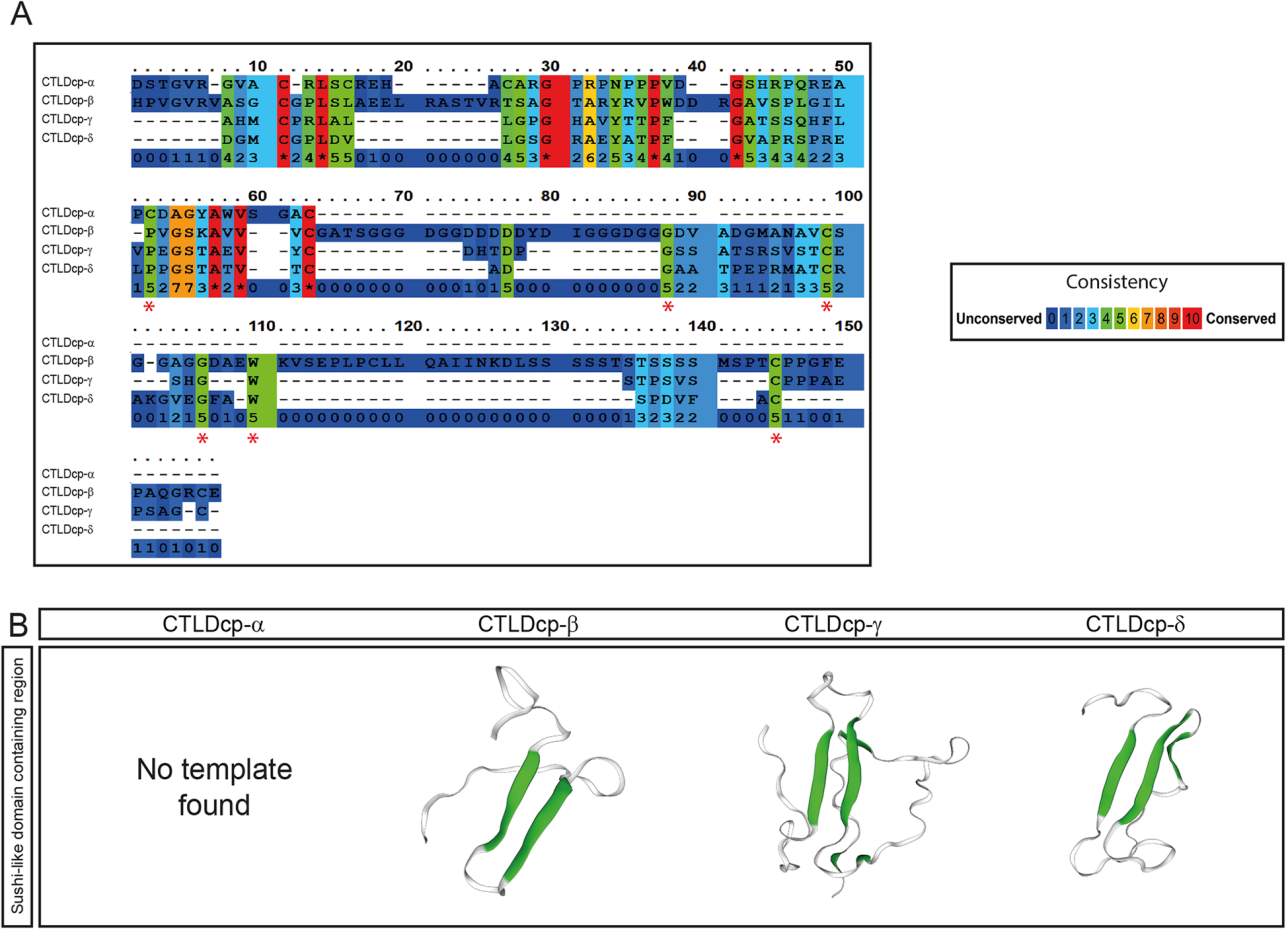
Identification of the sushi-like domain in Petromyzon marinus CTLDcps. **A** Amino acid alignment of the predicted sushi-like domain-containing region of *P. marinus* CTLDcp-α, -β, -γ and -δ. Domain conservation, defined by a consistency score, is color-coded. Red asterisks indicate critical residues of the sushi-like domain lost by the CTLDcp-α. **B** SWISS-MODEL results of the sushi-like domain reconstruction are displayed as 3D cartoon models. For CTLDcp-α the program failed to predict any tertiary structure

To understand the nature of *P. marinus* CTLDcps, we wondered if *P. marinus* proteins might share important features with their gnathostome counterparts. The presence of a sushi-like domain is a hallmark of CTLDcps and important for CTLDcps protein–protein interaction. Importantly, this extracellular motif (composed by ∼60 amino acid residues) is characterized by conserved tryptophan, glycine, proline, and cysteine residues [25]. Therefore, we retrieved the protein region bearing the sushi domain (see Analysis of the group XIV CTLDcps sushi-like domain in Petromyzon marinus and Protein domain identification and Ka/Ks ratio), and performed protein alignment and modeling prediction analyses of *P. marinus* proteins (Fig. 5). Protein alignment showed that one of the two CTLDcps found on chromosome 53 (CTLDcp-α) presented a short amino acid sequence as well as lacked key amino acid residues important for the sushi-like domain structure (red asterisks, Fig. 5A). This result was further substantiated by SWISS-MODEL, which failed to predict domain folding only for CTLDcp-α (Fig. 5B).

### Protein domain analysis of gnathostome CTLDcps

**Fig. 6:**
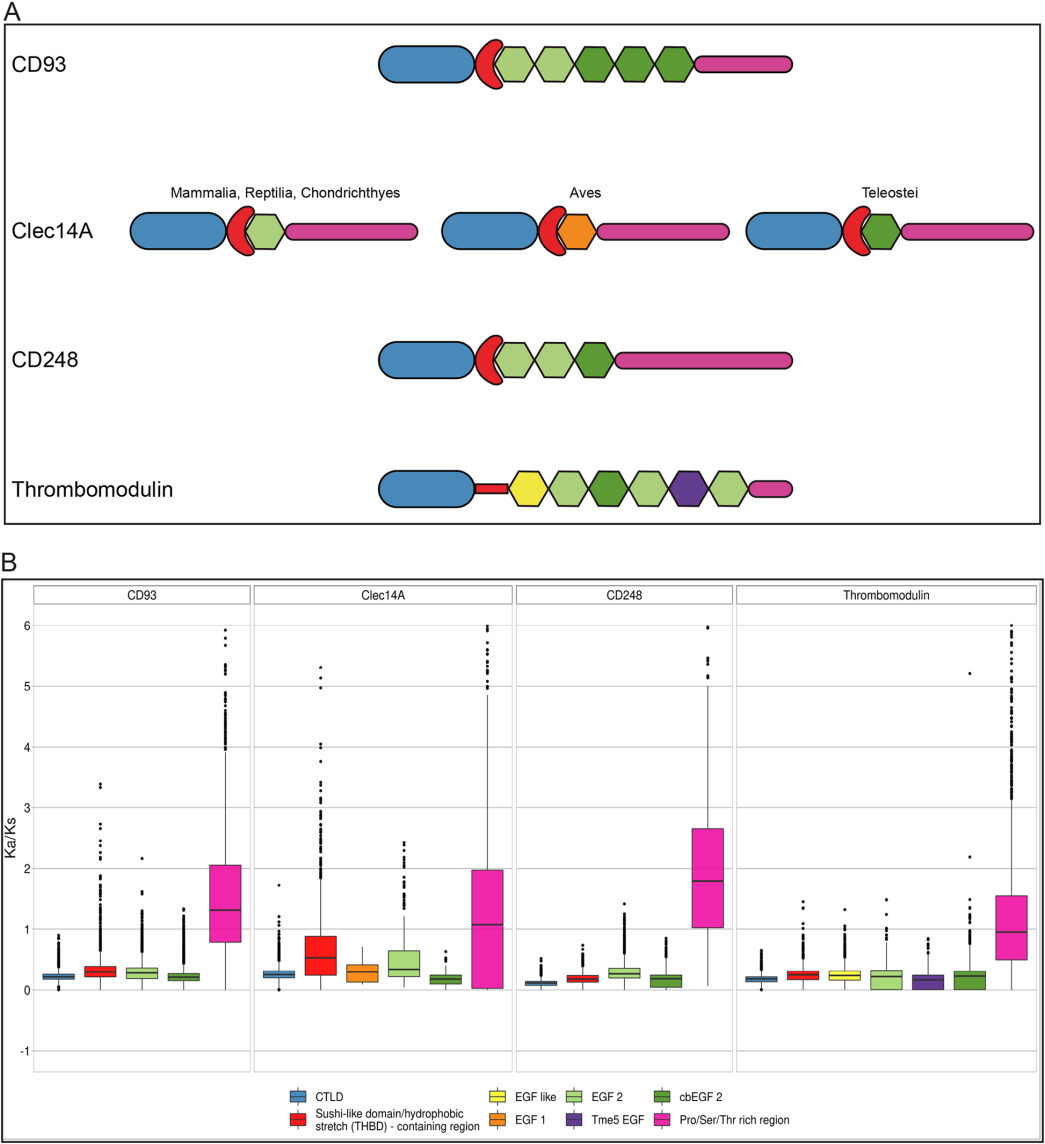
Evolutionary rate analysis of extracellular protein domains of the group XIV of CTLDcps. **A** Schematic representation of the group XIV extracellular protein domains. Each domain is color-coded as in **B**. **B** Ka/Ks ratio of each protein domain was calculated for all the gnathostome taxon using one representative species *per* order. Box plots showing the median and representing each domain are color-coded: CTLD (light blue), sushi-like domain/hydrophobic stretch (red), Pro/Ser/Thr rich (purple). EGF-like domains were dividend in different subgroups based on their sequence signatures and annotated as: EGF-like (yellow), EGF 1 (orange), EGF 2 (light green), cbEGF 2 (calcium-binding EGF, dark green), Tme5 EGF (Thrombomodulin-specific EGF, violet). The y-axis represents the non-synonymous (Ka) over synonymous (Ks) substitution ratio. Ka/Ks = 1 indicates neutral evolution. Ka/Ks ≤ 1 indicates purifying selection, Ka/Ks > 1 indicates positive selection. Pairwise comparison of each representative species *per* order (CD93 *n* = 71, Clec14A *n* = 55, CD248 *n* = 55 and Thrombomodulin *n* = 67). Black dots indicate outliers

Due to the importance of each CTLDcp domain in carrying out multiple and distinct functions, we sought to analyze in depth domain composition and study codon selection across gnathostome species. To do that, we collected amino acid sequences from one representative species *per* vertebrate order and identified protein domains using Batch-CD Search [24]. Despite clearly identifying the CTLD, Batch-CD Search failed to predict the sushi-like domain, the Pro/Ser/Thr rich region and part of the EGF-like domains, whose proper identification required manual curation (see Protein domain identification and Ka/Ks ratio). Protein extracellular domains (except for the EGF-like domain subtype) of each CTLDcp showed conservation of number and order across the vertebrate taxa and were then schematically represented in Fig. 6A. Interestingly, the main difference in domain composition was ascribable to EGF-like domains. In detail, while EGF 2 and cbEGF are present in all the CTLDcps, the EGF 1 is specific of the only Clec14A bird clade, whereas EGF-like and Tme5 EGF are distinctive of Thrombomodulin (Fig. 6A). To gain insights into the evolutionary rate of each protein domain, the nonsynonymous (Ka) over synonymous (Ks) ratio was estimated by comparing CDS sequences. As expected, the CTLD and the EGF-like domains of all the proteins showed a Ka/Ks ratio < 1 indicating a tendency to undergo purifying selection (Fig. 6B). Similarly, the sushi-like domain-containing region revealed a Ka/Ks < 1, although higher in Clec14A compared to CD93 and CD248. Curiously, despite Thrombomodulin does not present a proper sushi-like domain, we found this region highly conserved (Fig. 6B). Similarly, a purifying selection was also discovered in all the examined EGF domains of all proteins. In contrast, the Pro/Ser/Thr rich region showed a Ka/Ks ratio > 1, indicating positive selection (Fig. 6B, violet plots).

### CD93 and Thrombomodulin show tyrosine phosphorylation *consensuses* in their cytoplasmic domain

**Fig. 7:**
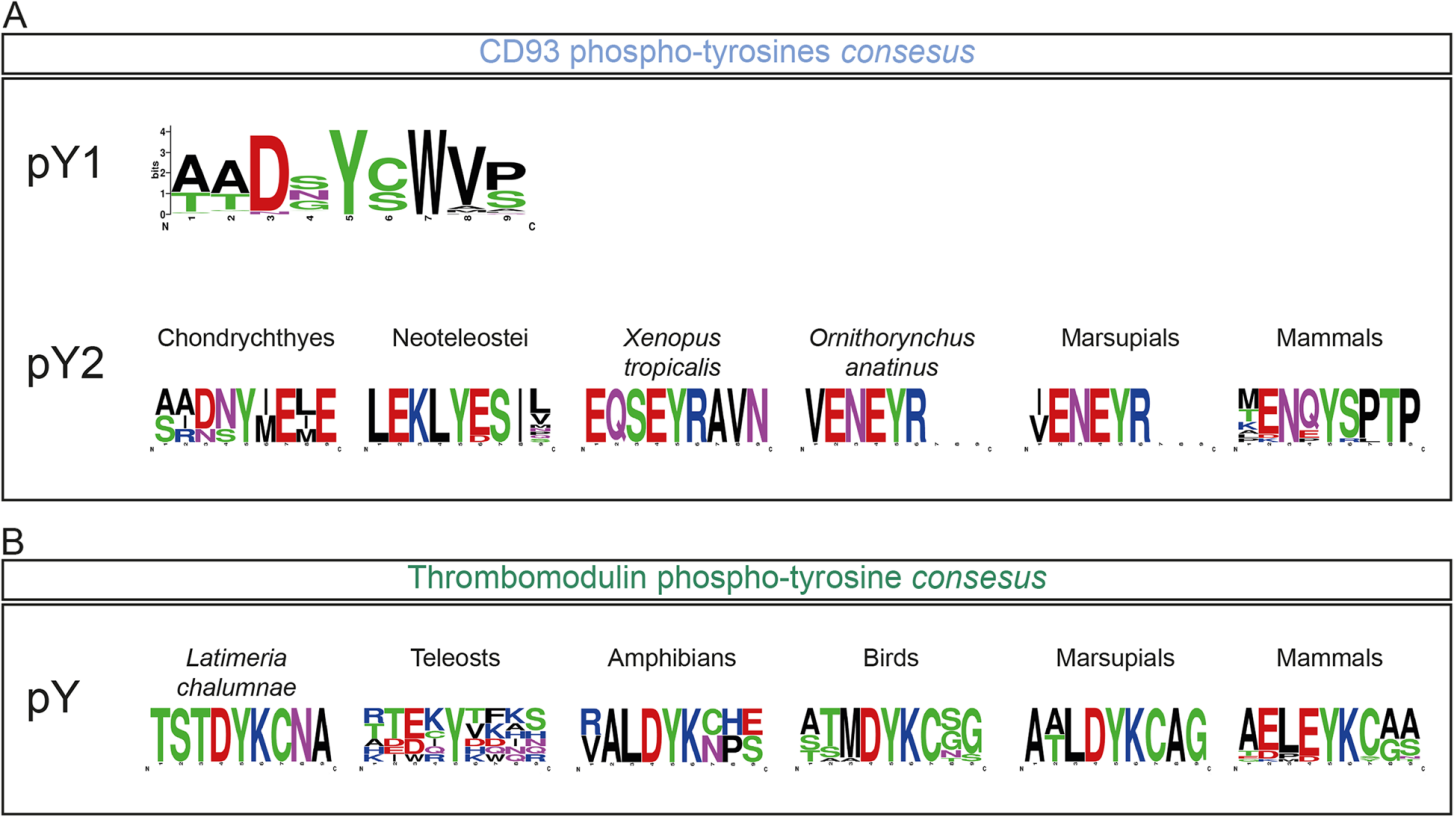
Identification of phospho-tyrosine consensuses in CD93 and Thrombomodulin. CD93 and Thrombomodulin phospho-tyrosine *consensuses* analysis. Positions of the amino acids are represented from the N- to the C-*terminus* in the x-axis and chemical properties of residues are color-coded. **A** Upper panel, CD93 phospho-tyrosine 1 (pY1) common *consensus* of all the  vertebrate clade (*n* = 68) residue frequencies are shown as probability rendering and expressed in bits. Lower panel, visualization of the CD93 second phospho-tyrosine (pY2) *consensus* in six vertebrate taxa. Sequences are shown as entropy rendering to emphasize motif’s information content. Number of species used for the analysis: Chondrichthyes *n* = 4, Neoteleostei *n* = 27, Marsupials *n* = 2 and Mammals *n* = 13. **B** Thrombomodulin phospho-tyrosine residue (pY) assessed independently for six vertebrate taxa: Teleosts *n* = 7, Amphibians *n* = 2, Birds *n* = 14, Marsupials *n* = 2 and Mammals *n* = 19. Sequences are shown as entropy rendering to emphasize motif’s information content

Tyrosine phosphorylation of transmembrane proteins is often a critical post-translational modification in regulating cell signaling and protein recycling. We therefore attempted to predict tyrosine phosphorylation of the entire group XIV using the NetPhos3.1 server. Analysis of the Clec14A sequence showed a lack of tyrosine residues in the cytoplasmic domain, while no significant predictions were found for CD248, despite the presence of a conserved tyrosine residue in the cytoplasmic domain. Consistent with previous findings on HUVECs [46], CD93 showed two tyrosine phosphorylation sites. Noteworthy, the first *consensus* was ubiquitous in all the vertebrate classes (Additional file 2: Supplementary Fig. 2) and showed a common amino acidic pattern (Fig. 7A, upper panel). Conversely, the second *consensus* differed between groups of gnathostomes and in fishes it was only found in the Neoteleostei clade (Fig. 7A, lower panel). Interestingly, a tyrosine *consensus* in the cytotail of Thrombomodulin was predicted as a possible phosphorylation site in six different vertebrate classes, resembling a common amino acid pattern, with the exception of fishes (Fig. 7B).

## Discussion

In this study, we investigated the evolution and the protein domain composition of the group XIV of CTLDcps in modern vertebrates. Here, we demonstrated that these proteins duplicated from a common ancestor and gained insights on their evolution in cyclostomes and gnathostomes. Moreover, we carefully analyzed CTLDcp domains by investigating their codon substitution rate and carefully identified and categorized EGF-like domains of several gnathostome species.

**Fig. 8:**
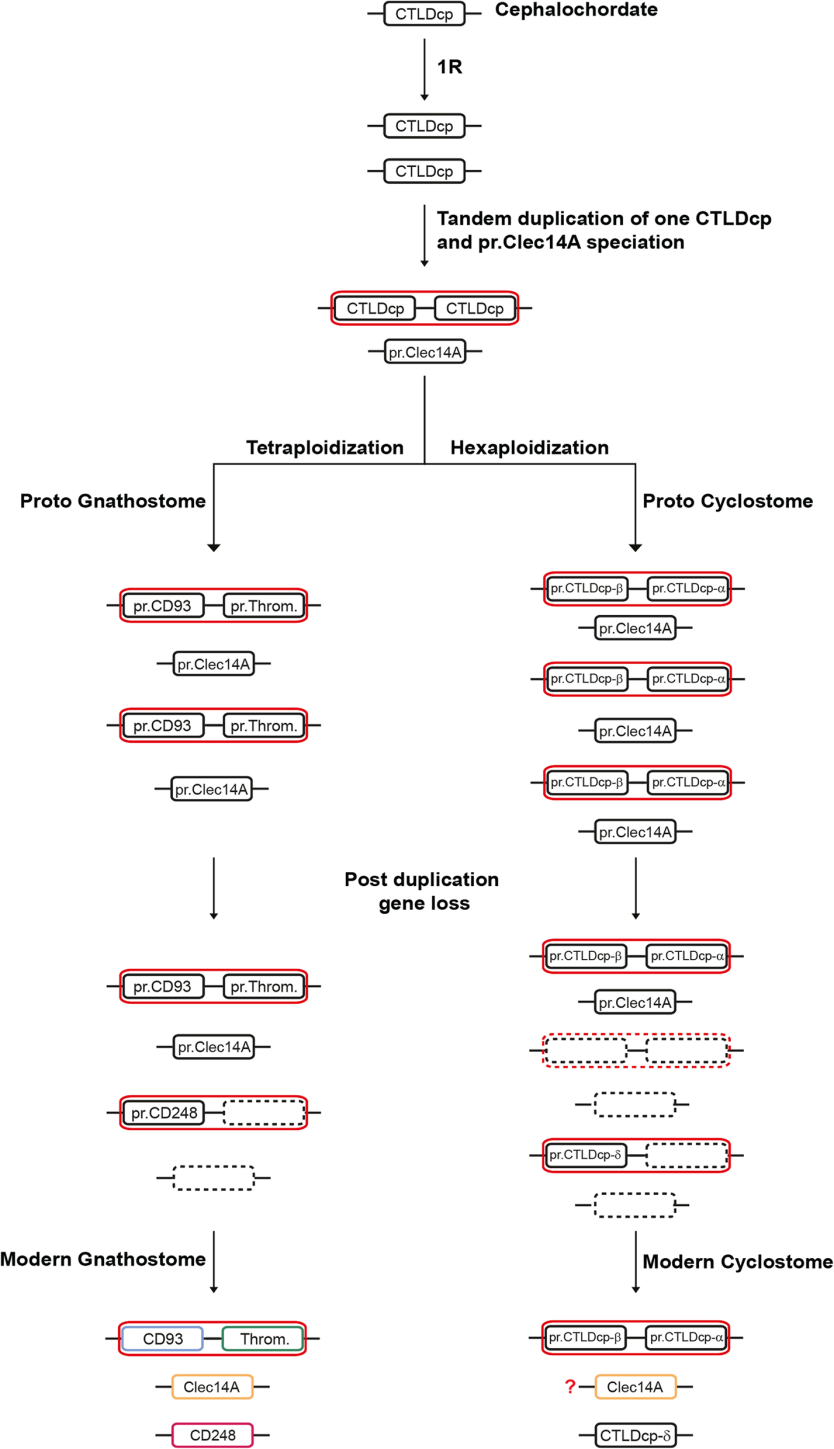
Proposed evolutionary scheme of the group XIV of CTLDcps. Evolutionary scheme of CTLDcps considering that *Clec14A* speciated before the divergence of gnathostomes and cyclostomes. ‘’pr.’’ indicates ‘’proto’’

Combining both phylogenetic and synteny-based approaches, we demonstrated that gnathostome proteins belonging to the group XIV of CTLDcps are paralogs and descend from a common ancestor. Here, we also attempted to unravel the phylogenetic relationships between gnathostome and cyclostome CTLDcps. Based on our results, we inferred an evolutionary scheme where a proto-vertebrate evolved two CTLDcp genes after one WGD event occurred in the ancestral cephalochordate. Since, in both gnathostomes (*CD93* and *Thrombomodulin*) and *P. marinus* (*CTLDcp*-α and -β), a pair of CTLDcps lies on the same chromosome only a few nucleotides apart, we assumed that their ancestral gene evolved by a tandem duplication event, likely due to asymmetric crossing-over as happened from other tandem duplicated genes [47]. Interestingly, analysis of *P. marinus* proteins revealed that one of the two tandem paralog genes lacks the sushi-like domain, suggesting that, before the divergence of gnathostomes and cyclostomes, the common ancestor of Thrombomodulin and CTLDcp-α lost key amino acids necessary for the folding of the sushi-like domain. From three ancestral CTLDcps in the proto-vertebrate, independent polyploidization of the proto-gnathostome and proto-cyclostome has generated multiple copies of CTLDcp genes, of which four have been retained in the majority of modern species (Fig. 8). Although our phylogenetic reconstruction did not show unambiguous orthology relationships between cyclostome and gnathostome CTLDcps, one of the alternative topologies identified *CTLDcp*-γ of *P. marinus* as ortholog of the gnathostome *Clec14A*. Interestingly, analysis of numerous CTLDcp orthologs revealed that each CTLDcp presents a statistically significant typical length (Additional file 3: Supplementary Fig. 3) with *Clec14A* being the shortest and having retained one or none EGF-like domain. Importantly, *P. marinus* CTLDcp-γ, showed a loss of the EGF-like domains, which accounts for its short protein sequence. This data suggests that *Clec14A* might have speciated before the separation of proto-gnathostomes from proto-cyclostomes. Moreover, a scenario where *Clec14A* speciated in the proto-vertebrate would implicate that the gnathostome *CD248* evolved from a *proto-CD93 locus* that underwent *proto-Thrombomodulin* loss. This assumption is substantiated by the fact that, according to our phylogenetic reconstructions, *CD93* is sister to *CD248* in gnathostomes (Figs. 2 and 4). In a similar fashion, the ortholog of *CTLDcp-*δ in cyclostomes might have arisen following a gene loss of one proto-CTLDcp-α copy (Fig. 8). Our findings are in line with the current view of evolution and divergence of modern cyclostomes and gnathostomes which suggests that, after a first WGD event, gnathostomes and cyclostomes underwent tetraploidization and hexaploidization respectively [3]. Noteworthy, synteny analyses of gnathostome CTLDcp genes showed that, while *CD93* and *Thrombomodulin* were found in all the representative vertebrate classes, a gene loss of *CD248* in birds and *Clec14A* in monotremes and marsupials occurred *via* different mechanisms. Indeed, gene *loci* analyses showed that *CD248* loss was due to a dismantling of its bearing chromosome since all the syntenic gene families were either lost or moved on other chromosomes. On the other hand, *Clec14A* loss was probably due to gene deletion or pseudogenization, and independently on chromosome rearrangements since its *locus* was maintained unaltered.

Since a preponderant role is attributed to the ECD in mediating group XIV protein function, we analyzed in depth ECD composition and investigated domain conservation by analyzing the mutation rate of each protein domain in gnathostomes. Consistent with the importance of the CTLD in maintaining protein folding and function [4], we found it to be deeply conserved in all the group XIV proteins and across the entire gnathostome clade. Interestingly, the region containing the sushi-like domain was also found to be highly conserved. Indeed, together with the CTLD, this relatively short region bears critical amino acid residues that mediate CD93, Clec14A and CD248 binding to the ECM protein MMRN2 [6, 48]. To our surprise, although Thrombomodulin lost key amino acids required for the folding of a proper sushi-like domain, we found the region comprised between the CTLD and the first EGF-like domain as highly conserved, indicating an important role in maintaining a functional protein structure. Our analysis pointed out some critical differences in the composition and organization of EGF-like domains between proteins. Although the EGF-like domains are annotated and have been previously described for the human and mouse proteins [9], some key differences in their composition have been overlooked. Except for the first Thrombomodulin EGF-like domain, all the other group members display the typical features of the complement C1r-like EGF domains (cEGF). Some EGF-like domains were found to bear a hydroxylation *consensus* of an asparagine residue as well as negatively charged residues at their N-*termini*, known to be critical in mediating calcium binding in accordance to the current PROSITE annotation [36] and previous studies [37]. Importantly, Clec14A showed different types of EGF-like domains between classes. Indeed, although being both cEGF subtypes [37], we found that in birds the EGF-like domain displayed the typical EGF 1 subtype signature, while mammals, reptiles, sharks and fishes showed the EGF 2 signature instead. In addition, in fishes, the Clec14A EGF-like domain bears a calcium-binding motif, supporting the idea of a species-specific evolution of different EGF-like domains in Clec14A. Regarding the Pro/Ser/Thr rich region, we found that it is characterized by a high mutation rate and subjected to positive selection. However, we found that in all species this region was highly enriched in amino acid residues that can be O-linked glycosylated (see Additional files 8, 9, 10 and 11: Supplementary files 1–4), suggesting that the overall O-linked sugar moiety might not be impaired. Our characterization of the CTLDcp domains was not limited on the ECD, as we also extended our analysis to the cytoplasmic region in the attempt to predict tyrosine phosphorylation motifs. Although Clec14A and CD248 showed no prediction of phosphorylable tyrosines, CD93 and Thrombomodulin were predicted to be tyrosine phosphorylated. According to previous observations [10, 46], for CD93 two *consensuses* of tyrosine phosphorylation were found in its intracellular domain. It is important to underline that, while all species were found to share the first tyrosine phosphorylation motif with a common *consensus*, only a few groups showed a second. Interestingly, the second *consensus* was found to be different across species, and in fishes was predicted solely for the Neoteleostei clade. This result leads to the hypothesis that differently from the first tyrosine residue, which might have appeared in the early evolution of CD93, the second phosphorylation site evolved lately and independently for each vertebrate group. Finally, although a tyrosine phosphorylation of Thrombomodulin has never been demonstrated experimentally, our analysis predicted a conserved phosphorylation *consensus* across different gnathostome classes. Our observations concerning the protein domain composition showed important class-specific synapomorphies. If on one hand, our study on protein alignments shows that each CTLDcp is highly conserved within classes, on the other, it points out critical inter-class differences. These results gain increasing relevance when considering the employment of animal models. In fact, although of valuable importance, the translation of results from model organisms that diverged several million years ago from humans needs to take into account subtle species-specific differences that might be of critical importance in inferring protein function. As an example, *Danio rerio* (zebrafish) is largely used as model to study angiogenesis and vascular dynamics and, specifically, has been employed to study the role of CD93 and Clec14A in angiogenesis [49]. As demonstrated by our analyses, the mammalian orthologs of CD93 and Clec14A in *D. rerio* showed differences in the protein domain composition. Indeed, CD93 in *D. rerio* only contains one phosphorylable tyrosine residue in its cytoplasmic domain compared to the two of mammals, suggesting relevant differences between fish and mammalian signaling pathways. Likewise, in fishes Clec14A showed a calcium-binding EGF-like domain which is not present in mammals and might entail important functional repercussions.

## Conclusions

In conclusion, using a phylogenetic and synteny-based approach, our study provides insights on the evolution of the group XIV of CTLDcps in modern vertebrates. Moreover, our analysis on protein domains allowed a visualization of the evolutionary rate of each domain and proposed a protein-specific classification of EGF-like domains based on known sequence signatures. Taken together, our results might help to better interpret protein functions and underline differences of orthologous proteins between different animal classes.

## Supplementary Information

**Additional file 1: Supplementary Figure 1.** Alternative tree topologies. Each tree represents the constrained topology tested with 10,000 RELL replicates for different statistical methods (bp-RELL, p-KH, p-SH, c-ELW, p-AU) through IQ-TREE. Outcomes of each analysis is detailed in Supplementary Table 4.

**Additional file 2: Supplementary Figure 2.** Identification of the first CD93 phospho-tyrosine consensus in each group of vertebrates. CD93 first phospho-tyrosine consensus was retrieved by NetPhos3.1 and plotted using WebLogo2.8.2. The predicted *consensus* for each group of vertebrates is shown as entropy rendering abundance to emphasize motif’s information content. Number of species used for the analysis: Chondrichthyes *n* = 3, Teleosts *n* = 27, Amphibians *n* = 2, Reptiles *n* = 2, Birds *n* = 11, Marsupials *n* = 3 and Mammals *n* = 17.

**Additional file 3: Supplementary Figure 3.** Average protein length of group XIV CTLDcps. Protein length was calculated and plotted in the R environment v.3.6.3. Number of species used for the analysis: CD93 *n*= 98, Clec14A *n*= 64, CD248 *n*= 61 and Thrombomodulin *n*= 88. Bars show SD. ****P < 0.0001 One-way ANOVA test.

**Additional file 4: Supplementary Table 1.** List of protein (first sheet), CDS (second sheet), cDNA (third sheet) accession numbers used and genome accession number of *Lethenteron camtschaticum*, *Entosphenus tridentatus*,* Eptatretus burgeri *(fourth sheet).

**Additional file 5: Supplementary Table 2.** Gnathostome CTLDcps loci synteny. List of the syntenic genes laying on the *CD93/Thrombomodulin- *(first sheet), *CD248-* (second sheet) and *Clec14A-* (third sheet) -bearing chromosomes in gnathostomes. Positions of genes that moved away from the chromosome are specified. § represents not found genes. # represents genes with an unplaced scaffold. * indicates chromosome disruption.

**Additional file 6: Supplementary Table 3. ***P. marinus* gene synteny. Gene families laying on chromosome 6, 17 and 53 of *P. marinus* analyzed for conserved synteny with their gnathostome counterparts.

**Additional file 7: Supplementary Table 4.** Alternative topology test results. Eleven topologies tested with five different statistical methods (bp-RELL, p-KH, p-SH, c-ELW, p-AU). Tested tree topologies follow unconstrained and constrained trees reported in Supplementary Fig. 1. Highlighted rows are significant topologies.

**Additional file 8: Supplementary file 1.** CDS of one representative species *per* vertebrate order of CD93 extracellular domain.

**Additional file 9: Supplementary file 2.** CDS of one representative species *per *vertebrate order of Clec14A extracellular domain.

**Additional file 10: Supplementary file 3.** CDS of one representative species *per *vertebrate order of CD248 extracellular domain.

**Additional file 11: Supplementary file 4.** CDS of one representative species *per *vertebrate order of Thrombomodulin extracellular domain.

## Data Availability

The datasets analyzed during the current study are available in the NCBI database repository, https://www.ncbi.nlm.nih.gov/. Protein domain CDS of each CTLDcp in the different gnathostome orders can be found as additional files.

## Abbreviations

CDS: Coding sequence
cEGF: Complement C1r-like EGF domain
cbEGF: Calcium-binding EGF
CTLDcp: C-type lectin domain-containing protein
EC: Endothelial cells
ECD: Extracellular domain
EGF: Epidermal growth factor
FGFR1: Fibroblast growth factor receptor 1
MMRN2: Multimerin-2
ML: Maximum-Likelihood
sCD93: Soluble CD93
WGD: Whole Genome Duplication
