# Supplementary material for: A glimpse into the past: phylogenesis and protein domain analysis of the group XIV of C-type lectins in vertebrates
Source: BMC Genomics. 2022 Jun 4;23:420. doi: 10.1186/s12864-022-08659-6 (PMC9167495; doi:10.1186/s12864-022-08659-6)
Supplement: Supplementary file 1 — Additional file 1: Supplementary Figure 1. Alternative tree topologies. Each tree represents the constrained topology tested with 10,000 RELL replicates for different statistical methods (bp-RELL, p-KH, p-SH, c-ELW, p-AU) through IQ-TREE. Outcomes of each analysis is detailed in Supplementary Table 4. [file 12864_2022_8659_MOESM1_ESM.pdf]

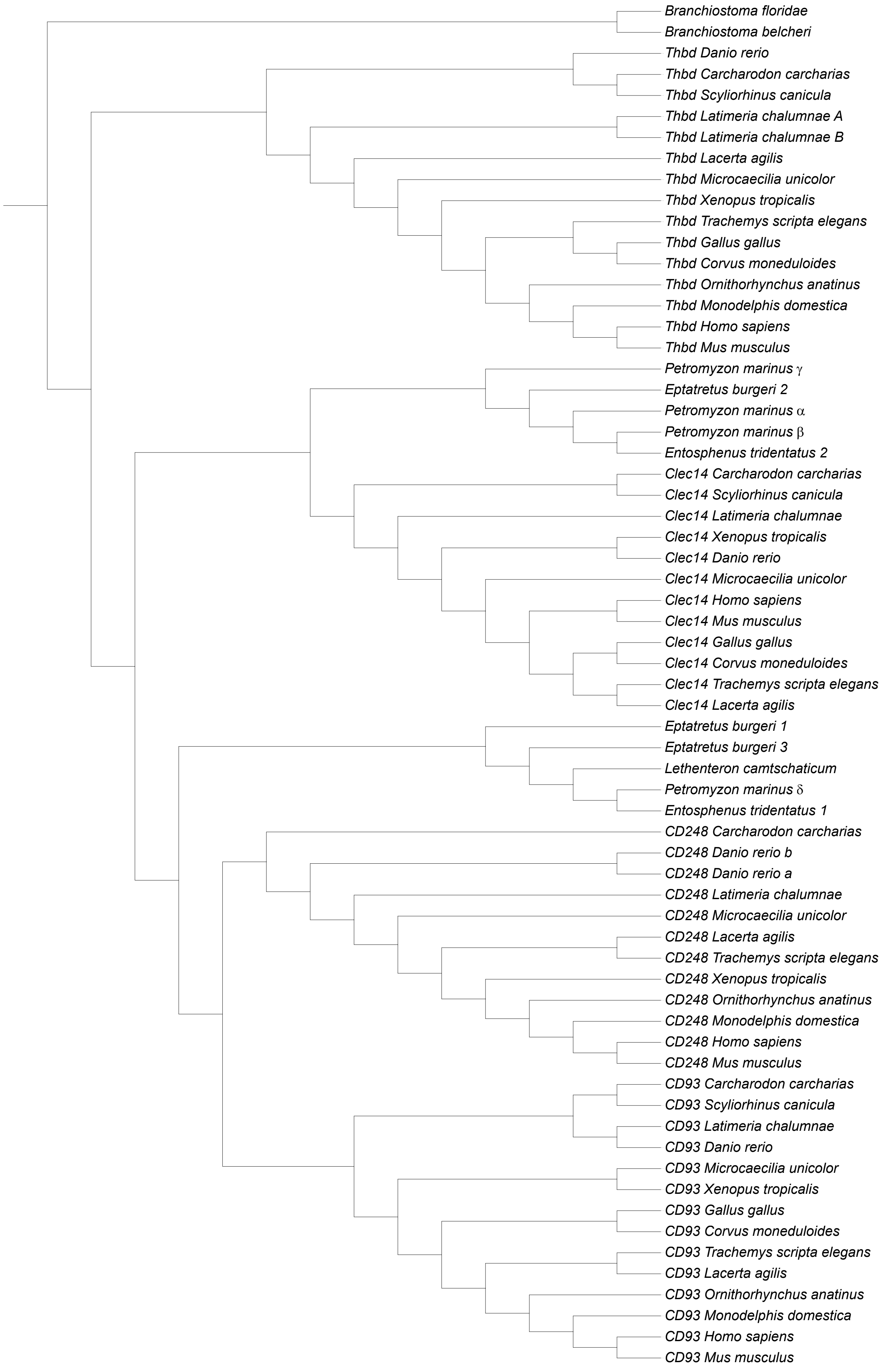

Unconstrained

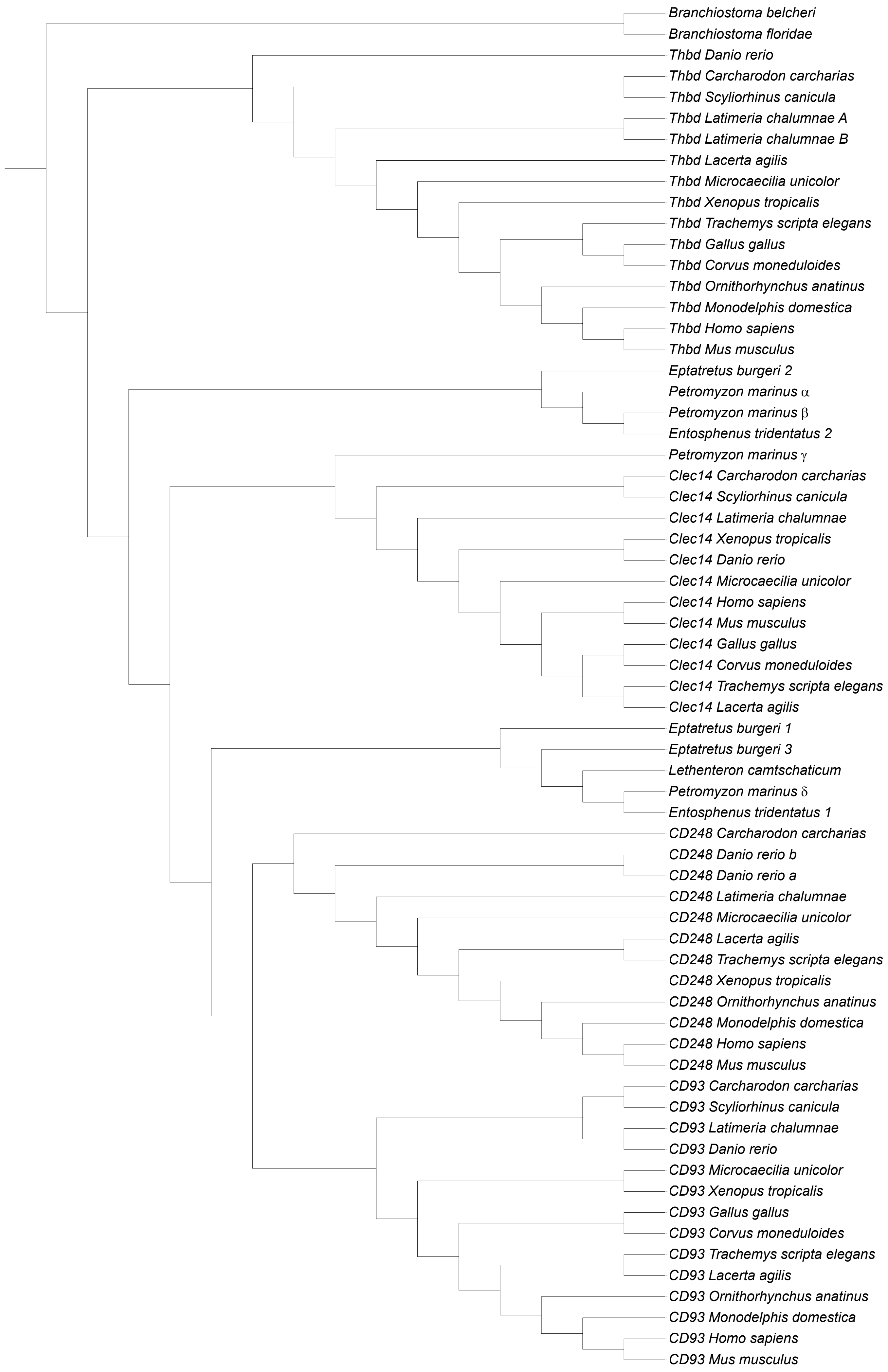
Branchiostoma belcheri
Branchiostoma floridae
Thbd Danio rerio
Thbd Carcharodon carcharias
Thbd Scyliorhinus canicula
Thbd Latimeria chalumnae A
Thbd Latimeria chalumnae B
Thbd Lacerta agilis
Thbd Microcaecilia unicolor
Thbd Xenopus tropicalis
Thbd Trachemys scripta elegans
Thbd Gallus gallus
Thbd Corvus moneduloides
Thbd Ornithorhynchus anatinus
Thbd Monodelphis domestica
Thbd Homo sapiens
Thbd Mus musculus
Eptatretus burgeri 2
Petromyzon marinus α
Petromyzon marinus β
Entosphenus tridentatus 2
Petromyzon marinus γ
Clec14 Carcharodon carcharias
Clec14 Scyliorhinus canicula
Clec14 Latimeria chalumnae
Clec14 Xenopus tropicalis
Clec14 Danio rerio
Clec14 Microcaecilia unicolor
Clec14 Homo sapiens
Clec14 Mus musculus
Clec14 Gallus gallus
Clec14 Corvus moneduloides
Clec14 Trachemys scripta elegans
Clec14 Lacerta agilis
Eptatretus burgeri 1
Eptatretus burgeri 3
Lethenteron camtschaticum
Petromyzon marinus δ
Entosphenus tridentatus 1
CD248 Carcharodon carcharias
CD248 Danio rerio b
CD248 Danio rerio a
CD248 Latimeria chalumnae
CD248 Microcaecilia unicolor
CD248 Lacerta agilis
CD248 Trachemys scripta elegans
CD248 Xenopus tropicalis
CD248 Ornithorhynchus anatinus
CD248 Monodelphis domestica
CD248 Homo sapiens
CD248 Mus musculus
CD93 Carcharodon carcharias
CD93 Scyliorhinus canicula
CD93 Latimeria chalumnae
CD93 Danio rerio
CD93 Microcaecilia unicolor
CD93 Xenopus tropicalis
CD93 Gallus gallus
CD93 Corvus moneduloides
CD93 Trachemys scripta elegans
CD93 Lacerta agilis
CD93 Ornithorhynchus anatinus
CD93 Monodelphis domestica
CD93 Homo sapiens
CD93 Mus musculus

Topology 1

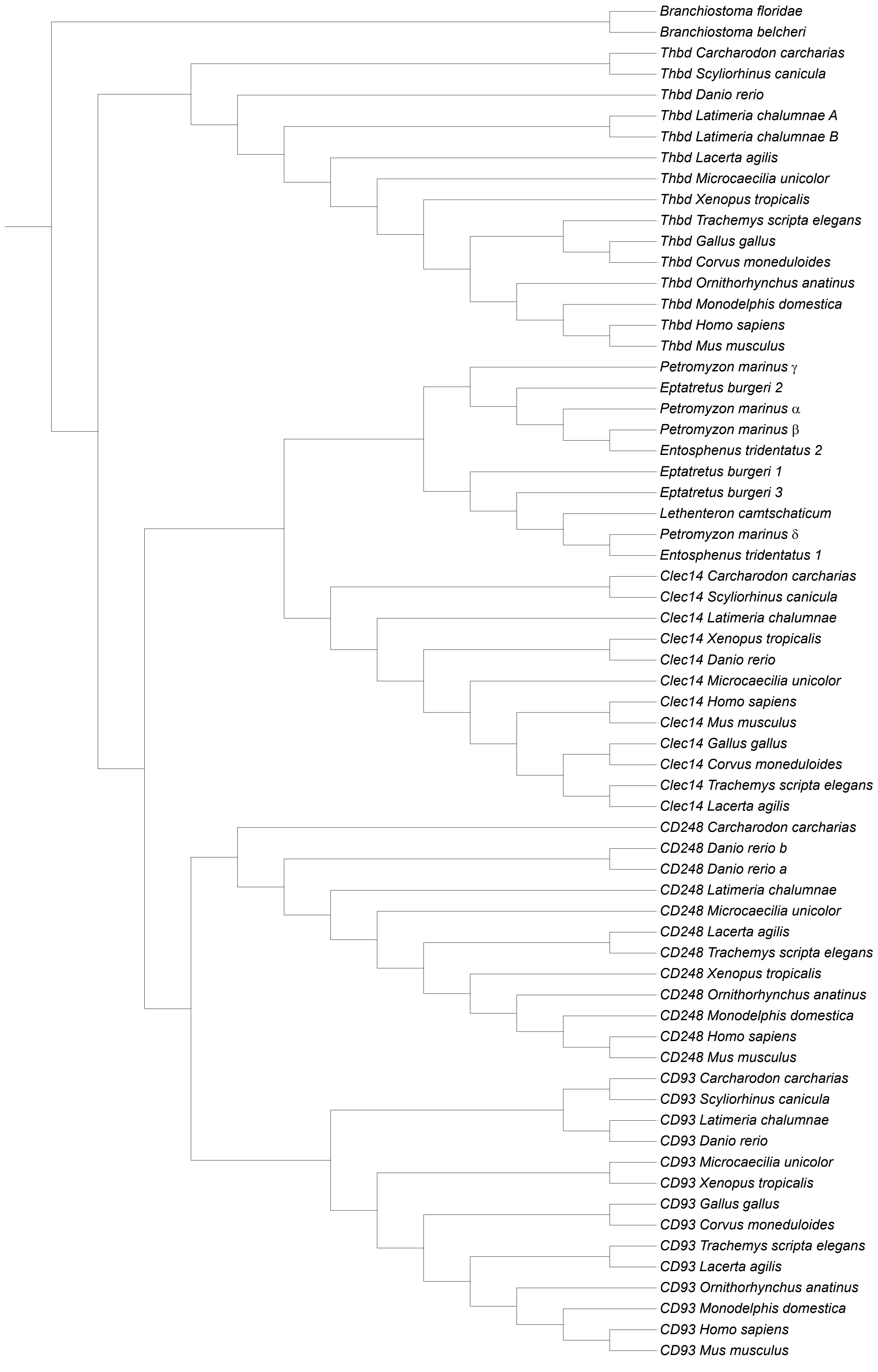
Branchiostoma floridae
Branchiostoma belcheri
Thbd Carcharodon carcharias
Thbd Scyliorhinus canicula
Thbd Danio rerio
Thbd Latimeria chalumnae A
Thbd Latimeria chalumnae B
Thbd Lacerta agilis
Thbd Microcaecilia unicolor
Thbd Xenopus tropicalis
Thbd Trachemys scripta elegans
Thbd Gallus gallus
Thbd Corvus moneduloides
Thbd Ornithorhynchus anatinus
Thbd Monodelphis domestica
Thbd Homo sapiens
Thbd Mus musculus
Petromyzon marinus γ
Eptatretus burgeri 2
Petromyzon marinus α
Petromyzon marinus β
Entosphenus tridentatus 2
Eptatretus burgeri 1
Eptatretus burgeri 3
Lethenteron camtschaticum
Petromyzon marinus δ
Entosphenus tridentatus 1
Clec14 Carcharodon carcharias
Clec14 Scyliorhinus canicula
Clec14 Latimeria chalumnae
Clec14 Xenopus tropicalis
Clec14 Danio rerio
Clec14 Microcaecilia unicolor
Clec14 Homo sapiens
Clec14 Mus musculus
Clec14 Gallus gallus
Clec14 Corvus moneduloides
Clec14 Trachemys scripta elegans
Clec14 Lacerta agilis
CD248 Carcharodon carcharias
CD248 Danio rerio b
CD248 Danio rerio a
CD248 Latimeria chalumnae
CD248 Microcaecilia unicolor
CD248 Lacerta agilis
CD248 Trachemys scripta elegans
CD248 Xenopus tropicalis
CD248 Ornithorhynchus anatinus
CD248 Monodelphis domestica
CD248 Homo sapiens
CD248 Mus musculus
CD93 Carcharodon carcharias
CD93 Scyliorhinus canicula
CD93 Latimeria chalumnae
CD93 Danio rerio
CD93 Microcaecilia unicolor
CD93 Xenopus tropicalis
CD93 Gallus gallus
CD93 Corvus moneduloides
CD93 Trachemys scripta elegans
CD93 Lacerta agilis
CD93 Ornithorhynchus anatinus
CD93 Monodelphis domestica
CD93 Homo sapiens
CD93 Mus musculus

Topology 2

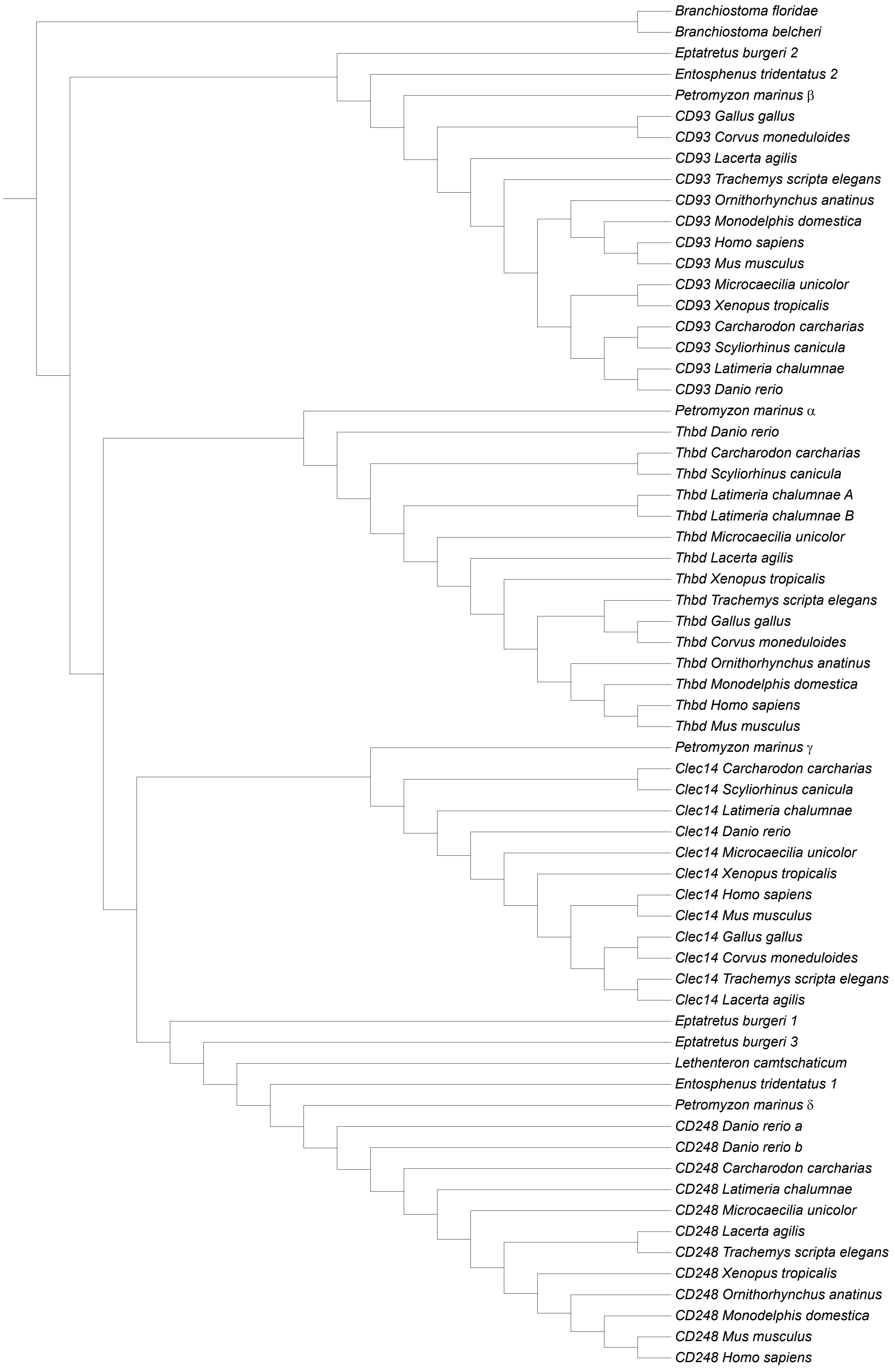
Branchiostoma floridae
Branchiostoma belcheri
Eptatretus burgeri 2
Entosphenus tridentatus 2
Petromyzon marinus β
CD93 Gallus gallus
CD93 Corvus moneduloides
CD93 Lacerta agilis
CD93 Trachemys scripta elegans
CD93 Ornithorhynchus anatinus
CD93 Monodelphis domestica
CD93 Homo sapiens
CD93 Mus musculus
CD93 Microcaecilia unicolor
CD93 Xenopus tropicalis
CD93 Carcharodon carcharias
CD93 Scyliorhinus canicula
CD93 Latimeria chalumnae
CD93 Danio rerio
Petromyzon marinus α
Thbd Danio rerio
Thbd Carcharodon carcharias
Thbd Scyliorhinus canicula
Thbd Latimeria chalumnae A
Thbd Latimeria chalumnae B
Thbd Microcaecilia unicolor
Thbd Lacerta agilis
Thbd Xenopus tropicalis
Thbd Trachemys scripta elegans
Thbd Gallus gallus
Thbd Corvus moneduloides
Thbd Ornithorhynchus anatinus
Thbd Monodelphis domestica
Thbd Homo sapiens
Thbd Mus musculus
Petromyzon marinus γ
Clec14 Carcharodon carcharias
Clec14 Scyliorhinus canicula
Clec14 Latimeria chalumnae
Clec14 Danio rerio
Clec14 Microcaecilia unicolor
Clec14 Xenopus tropicalis
Clec14 Homo sapiens
Clec14 Mus musculus
Clec14 Gallus gallus
Clec14 Corvus moneduloides
Clec14 Trachemys scripta elegans
Clec14 Lacerta agilis
Eptatretus burgeri 1
Eptatretus burgeri 3
Lethenteron camtschaticum
Entosphenus tridentatus 1
Petromyzon marinus δ
CD248 Danio rerio a
CD248 Danio rerio b
CD248 Carcharodon carcharias
CD248 Latimeria chalumnae
CD248 Microcaecilia unicolor
CD248 Lacerta agilis
CD248 Trachemys scripta elegans
CD248 Xenopus tropicalis
CD248 Ornithorhynchus anatinus
CD248 Monodelphis domestica
CD248 Mus musculus
CD248 Homo sapiens

Topology 3

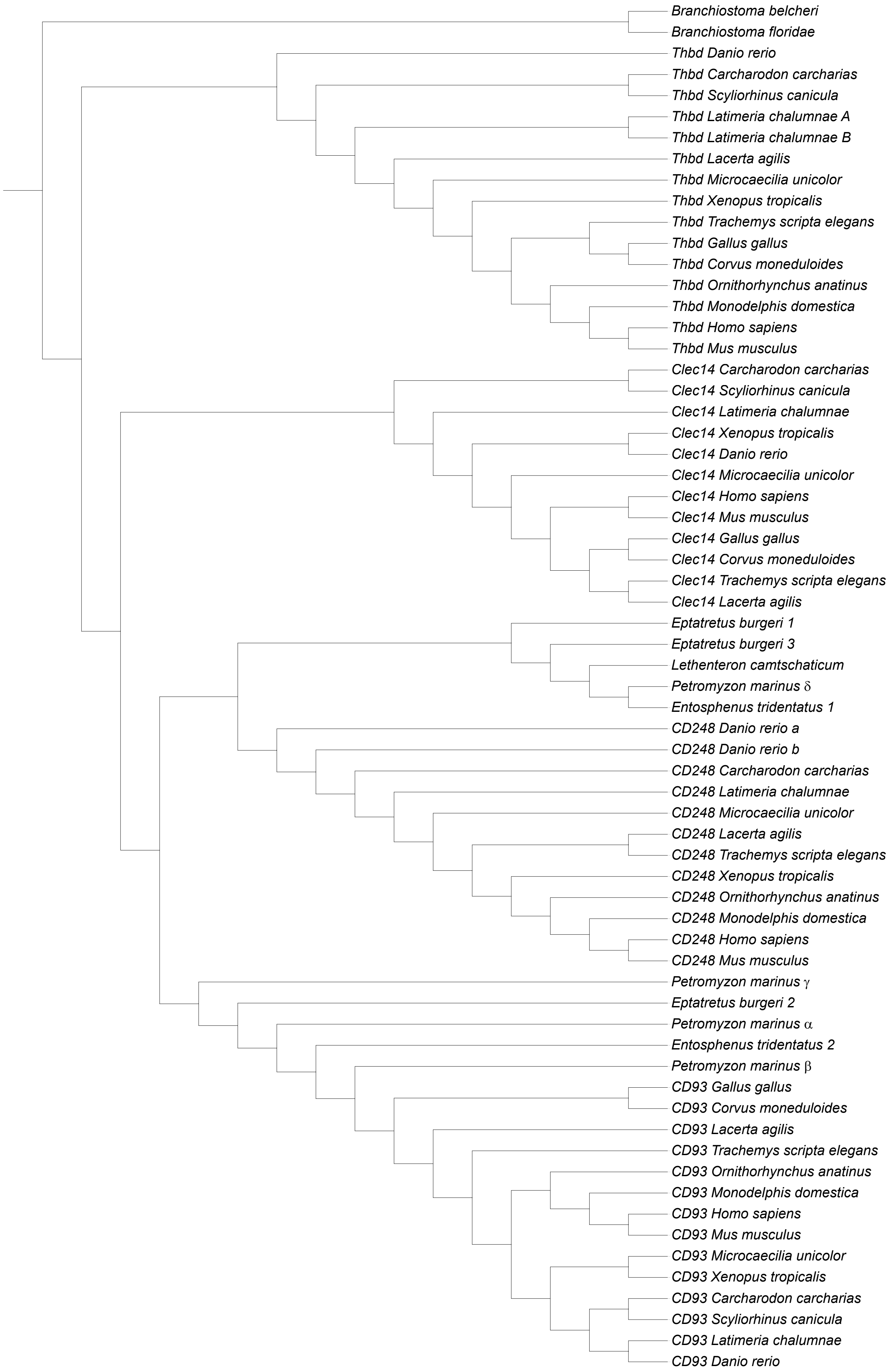
Branchiostoma belcheri
Branchiostoma floridae
Thbd Danio rerio
Thbd Carcharodon carcharias
Thbd Scyliorhinus canicula
Thbd Latimeria chalumnae A
Thbd Latimeria chalumnae B
Thbd Lacerta agilis
Thbd Microcaecilia unicolor
Thbd Xenopus tropicalis
Thbd Trachemys scripta elegans
Thbd Gallus gallus
Thbd Corvus moneduloides
Thbd Ornithorhynchus anatinus
Thbd Monodelphis domestica
Thbd Homo sapiens
Thbd Mus musculus
Clec14 Carcharodon carcharias
Clec14 Scyliorhinus canicula
Clec14 Latimeria chalumnae
Clec14 Xenopus tropicalis
Clec14 Danio rerio
Clec14 Microcaecilia unicolor
Clec14 Homo sapiens
Clec14 Mus musculus
Clec14 Gallus gallus
Clec14 Corvus moneduloides
Clec14 Trachemys scripta elegans
Clec14 Lacerta agilis
Eptatretus burgeri 1
Eptatretus burgeri 3
Lethenteron camtschaticum
Petromyzon marinus δ
Entosphenus tridentatus 1
CD248 Danio rerio a
CD248 Danio rerio b
CD248 Carcharodon carcharias
CD248 Latimeria chalumnae
CD248 Microcaecilia unicolor
CD248 Lacerta agilis
CD248 Trachemys scripta elegans
CD248 Xenopus tropicalis
CD248 Ornithorhynchus anatinus
CD248 Monodelphis domestica
CD248 Homo sapiens
CD248 Mus musculus
Petromyzon marinus γ
Eptatretus burgeri 2
Petromyzon marinus α
Entosphenus tridentatus 2
Petromyzon marinus β
CD93 Gallus gallus
CD93 Corvus moneduloides
CD93 Lacerta agilis
CD93 Trachemys scripta elegans
CD93 Ornithorhynchus anatinus
CD93 Monodelphis domestica
CD93 Homo sapiens
CD93 Mus musculus
CD93 Microcaecilia unicolor
CD93 Xenopus tropicalis
CD93 Carcharodon carcharias
CD93 Scyliorhinus canicula
CD93 Latimeria chalumnae
CD93 Danio rerio

Topology 4

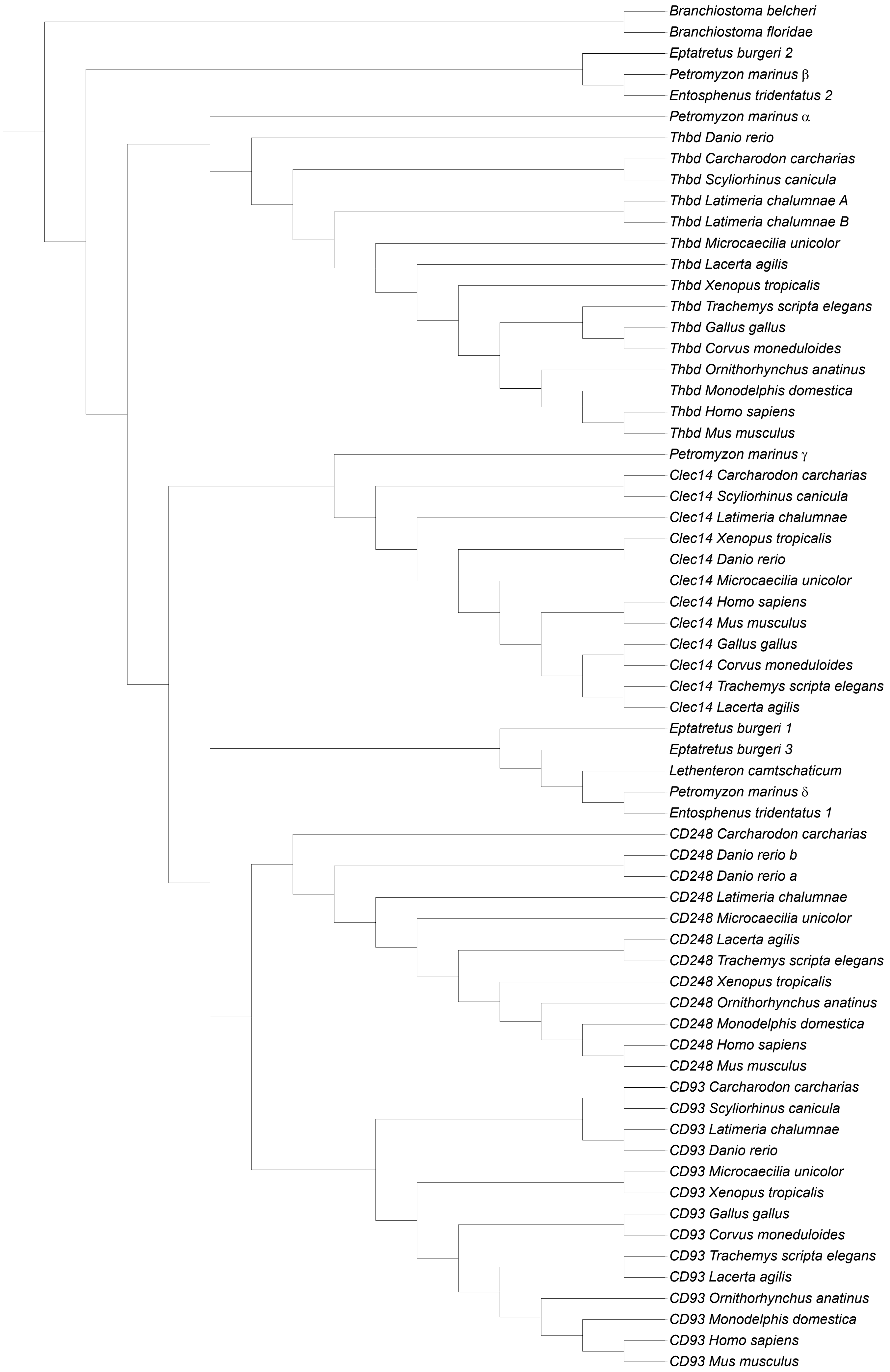
Branchiostoma belcheri
Branchiostoma floridae
Eptatretus burgeri 2
Petromyzon marinus β
Entosphenus tridentatus 2
Petromyzon marinus α
Thbd Danio rerio
Thbd Carcharodon carcharias
Thbd Scyliorhinus canicula
Thbd Latimeria chalumnae A
Thbd Latimeria chalumnae B
Thbd Microcaecilia unicolor
Thbd Lacerta agilis
Thbd Xenopus tropicalis
Thbd Trachemys scripta elegans
Thbd Gallus gallus
Thbd Corvus moneduloides
Thbd Ornithorhynchus anatinus
Thbd Monodelphis domestica
Thbd Homo sapiens
Thbd Mus musculus
Petromyzon marinus γ
Clec14 Carcharodon carcharias
Clec14 Scyliorhinus canicula
Clec14 Latimeria chalumnae
Clec14 Xenopus tropicalis
Clec14 Danio rerio
Clec14 Microcaecilia unicolor
Clec14 Homo sapiens
Clec14 Mus musculus
Clec14 Gallus gallus
Clec14 Corvus moneduloides
Clec14 Trachemys scripta elegans
Clec14 Lacerta agilis
Eptatretus burgeri 1
Eptatretus burgeri 3
Lethenteron camtschaticum
Petromyzon marinus δ
Entosphenus tridentatus 1
CD248 Carcharodon carcharias
CD248 Danio rerio b
CD248 Danio rerio a
CD248 Latimeria chalumnae
CD248 Microcaecilia unicolor
CD248 Lacerta agilis
CD248 Trachemys scripta elegans
CD248 Xenopus tropicalis
CD248 Ornithorhynchus anatinus
CD248 Monodelphis domestica
CD248 Homo sapiens
CD248 Mus musculus
CD93 Carcharodon carcharias
CD93 Scyliorhinus canicula
CD93 Latimeria chalumnae
CD93 Danio rerio
CD93 Microcaecilia unicolor
CD93 Xenopus tropicalis
CD93 Gallus gallus
CD93 Corvus moneduloides
CD93 Trachemys scripta elegans
CD93 Lacerta agilis
CD93 Ornithorhynchus anatinus
CD93 Monodelphis domestica
CD93 Homo sapiens
CD93 Mus musculus

Topology 5

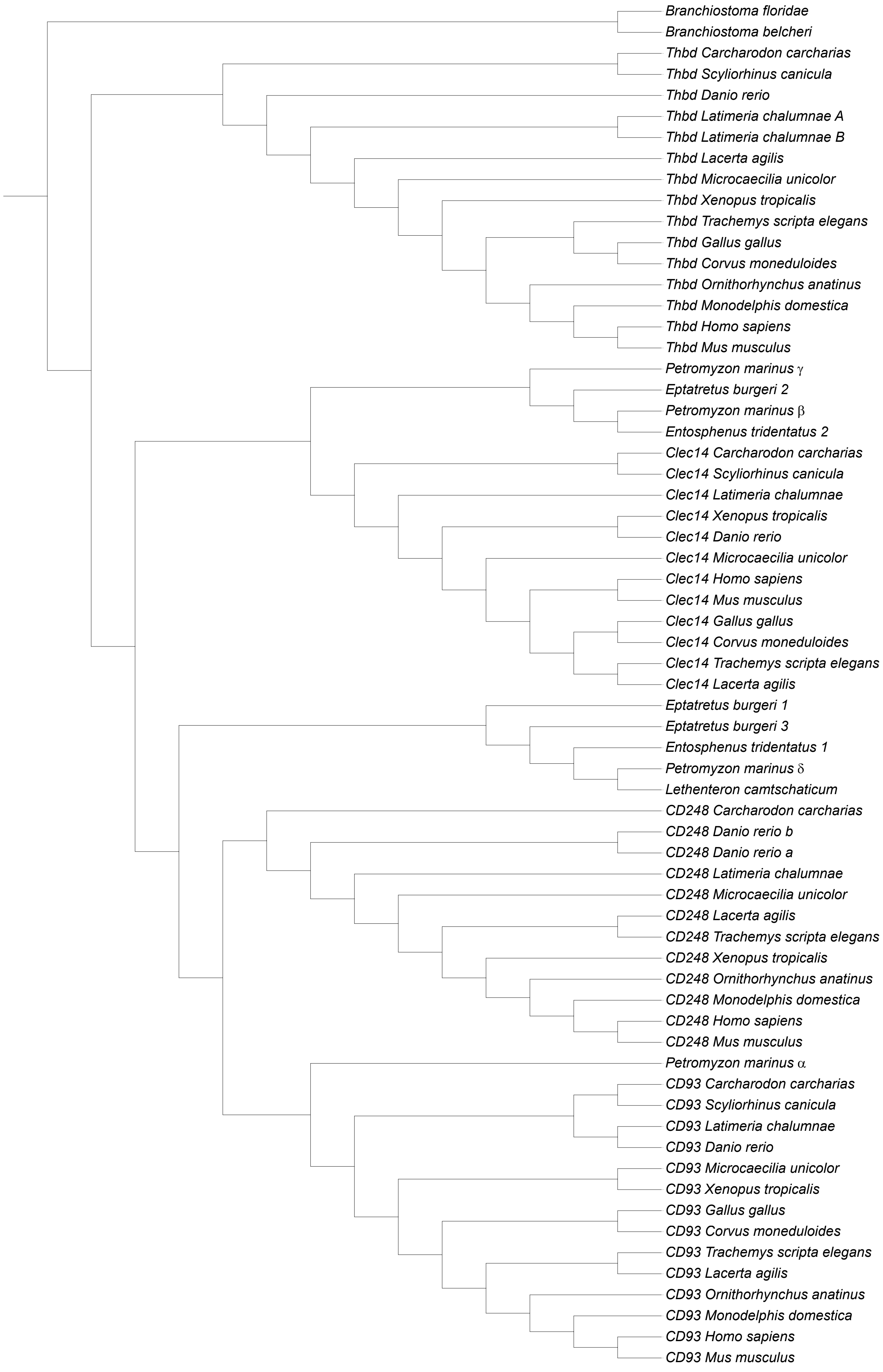
Branchiostoma floridae
Branchiostoma belcheri
Thbd Carcharodon carcharias
Thbd Scyliorhinus canicula
Thbd Danio rerio
Thbd Latimeria chalumnae A
Thbd Latimeria chalumnae B
Thbd Lacerta agilis
Thbd Microcaecilia unicolor
Thbd Xenopus tropicalis
Thbd Trachemys scripta elegans
Thbd Gallus gallus
Thbd Corvus moneduloides
Thbd Ornithorhynchus anatinus
Thbd Monodelphis domestica
Thbd Homo sapiens
Thbd Mus musculus
Petromyzon marinus γ
Eptatretus burgeri 2
Petromyzon marinus β
Entosphenus tridentatus 2
Clec14 Carcharodon carcharias
Clec14 Scyliorhinus canicula
Clec14 Latimeria chalumnae
Clec14 Xenopus tropicalis
Clec14 Danio rerio
Clec14 Microcaecilia unicolor
Clec14 Homo sapiens
Clec14 Mus musculus
Clec14 Gallus gallus
Clec14 Corvus moneduloides
Clec14 Trachemys scripta elegans
Clec14 Lacerta agilis
Eptatretus burgeri 1
Eptatretus burgeri 3
Entosphenus tridentatus 1
Petromyzon marinus δ
Lethenteron camtschaticum
CD248 Carcharodon carcharias
CD248 Danio rerio b
CD248 Danio rerio a
CD248 Latimeria chalumnae
CD248 Microcaecilia unicolor
CD248 Lacerta agilis
CD248 Trachemys scripta elegans
CD248 Xenopus tropicalis
CD248 Ornithorhynchus anatinus
CD248 Monodelphis domestica
CD248 Homo sapiens
CD248 Mus musculus
Petromyzon marinus α
CD93 Carcharodon carcharias
CD93 Scyliorhinus canicula
CD93 Latimeria chalumnae
CD93 Danio rerio
CD93 Microcaecilia unicolor
CD93 Xenopus tropicalis
CD93 Gallus gallus
CD93 Corvus moneduloides
CD93 Trachemys scripta elegans
CD93 Lacerta agilis
CD93 Ornithorhynchus anatinus
CD93 Monodelphis domestica
CD93 Homo sapiens
CD93 Mus musculus

Topology 6

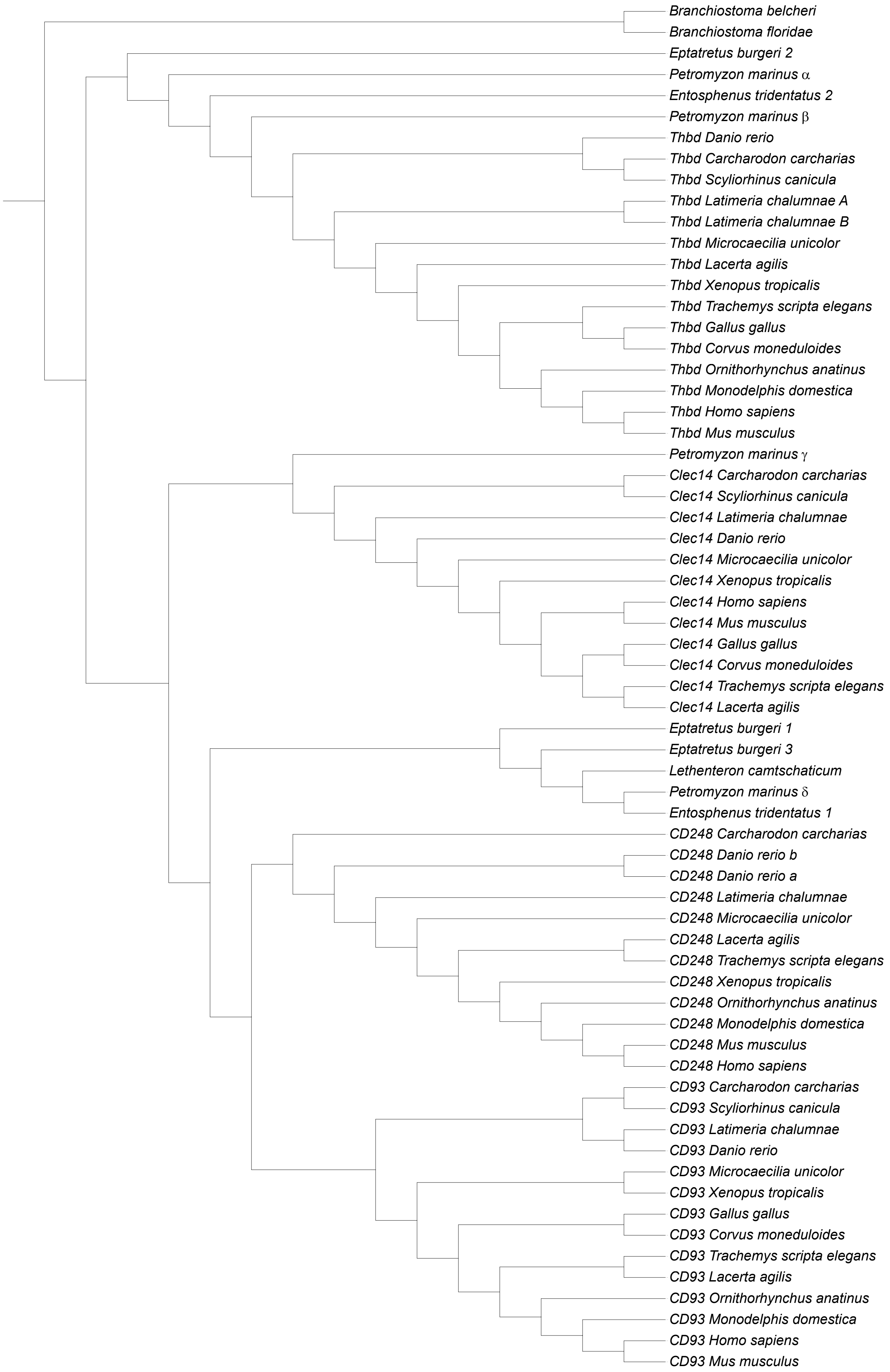
Branchiostoma belcheri
Branchiostoma floridae
Eptatretus burgeri 2
Petromyzon marinus α
Entosphenus tridentatus 2
Petromyzon marinus β
Thbd Danio rerio
Thbd Carcharodon carcharias
Thbd Scyliorhinus canicula
Thbd Latimeria chalumnae A
Thbd Latimeria chalumnae B
Thbd Microcaecilia unicolor
Thbd Lacerta agilis
Thbd Xenopus tropicalis
Thbd Trachemys scripta elegans
Thbd Gallus gallus
Thbd Corvus moneduloides
Thbd Ornithorhynchus anatinus
Thbd Monodelphis domestica
Thbd Homo sapiens
Thbd Mus musculus
Petromyzon marinus γ
Clec14 Carcharodon carcharias
Clec14 Scyliorhinus canicula
Clec14 Latimeria chalumnae
Clec14 Danio rerio
Clec14 Microcaecilia unicolor
Clec14 Xenopus tropicalis
Clec14 Homo sapiens
Clec14 Mus musculus
Clec14 Gallus gallus
Clec14 Corvus moneduloides
Clec14 Trachemys scripta elegans
Clec14 Lacerta agilis
Eptatretus burgeri 1
Eptatretus burgeri 3
Lethenteron camtschaticum
Petromyzon marinus δ
Entosphenus tridentatus 1
CD248 Carcharodon carcharias
CD248 Danio rerio b
CD248 Danio rerio a
CD248 Latimeria chalumnae
CD248 Microcaecilia unicolor
CD248 Lacerta agilis
CD248 Trachemys scripta elegans
CD248 Xenopus tropicalis
CD248 Ornithorhynchus anatinus
CD248 Monodelphis domestica
CD248 Mus musculus
CD248 Homo sapiens
CD93 Carcharodon carcharias
CD93 Scyliorhinus canicula
CD93 Latimeria chalumnae
CD93 Danio rerio
CD93 Microcaecilia unicolor
CD93 Xenopus tropicalis
CD93 Gallus gallus
CD93 Corvus moneduloides
CD93 Trachemys scripta elegans
CD93 Lacerta agilis
CD93 Ornithorhynchus anatinus
CD93 Monodelphis domestica
CD93 Homo sapiens
CD93 Mus musculus

Topology 7

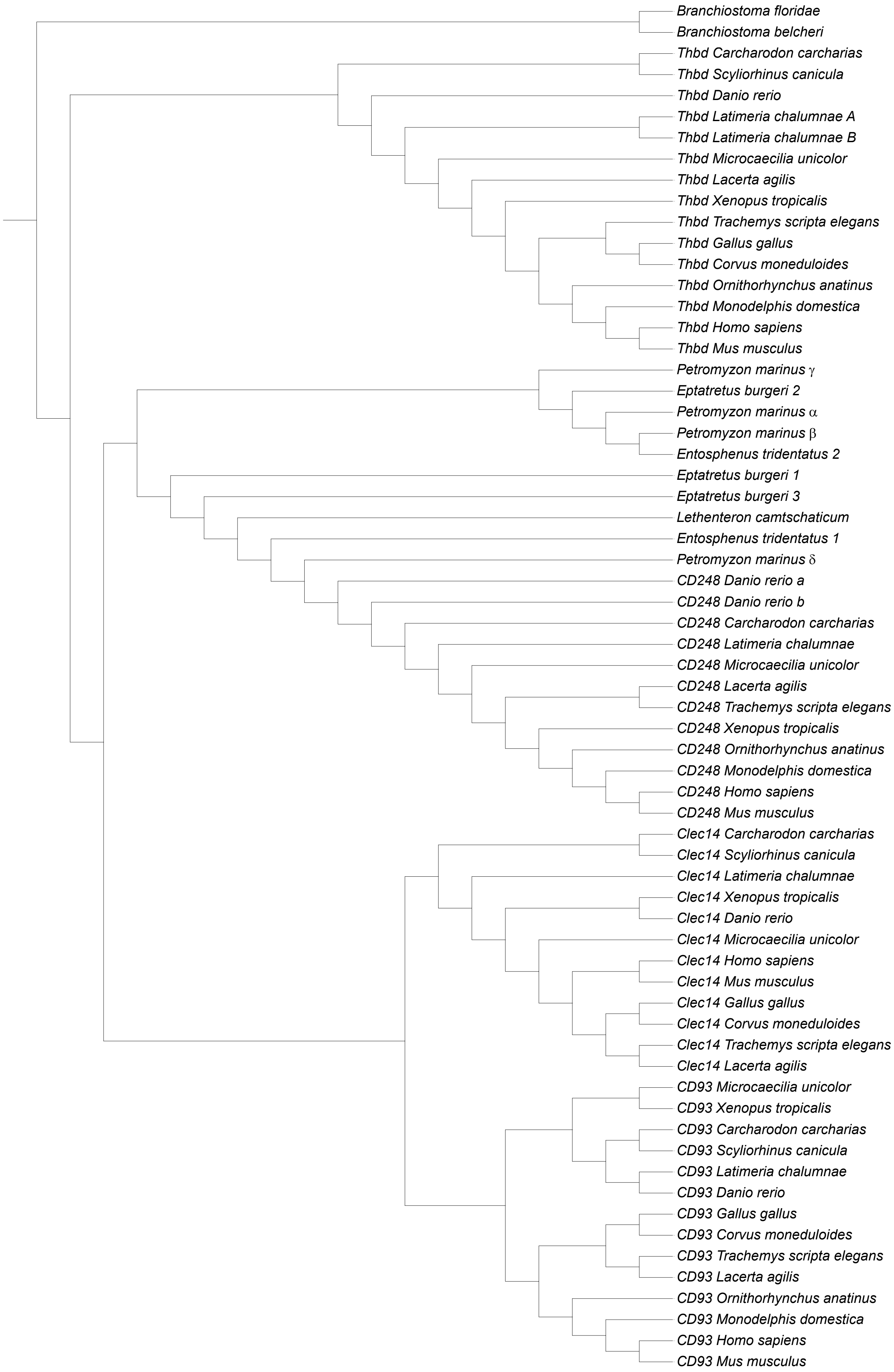
Branchiostoma floridae
Branchiostoma belcheri
Thbd Carcharodon carcharias
Thbd Scyliorhinus canicula
Thbd Danio rerio
Thbd Latimeria chalumnae A
Thbd Latimeria chalumnae B
Thbd Microcaecilia unicolor
Thbd Lacerta agilis
Thbd Xenopus tropicalis
Thbd Trachemys scripta elegans
Thbd Gallus gallus
Thbd Corvus moneduloides
Thbd Ornithorhynchus anatinus
Thbd Monodelphis domestica
Thbd Homo sapiens
Thbd Mus musculus
Petromyzon marinus γ
Eptatretus burgeri 2
Petromyzon marinus α
Petromyzon marinus β
Entosphenus tridentatus 2
Eptatretus burgeri 1
Eptatretus burgeri 3
Lethenteron camtschaticum
Entosphenus tridentatus 1
Petromyzon marinus δ
CD248 Danio rerio a
CD248 Danio rerio b
CD248 Carcharodon carcharias
CD248 Latimeria chalumnae
CD248 Microcaecilia unicolor
CD248 Lacerta agilis
CD248 Trachemys scripta elegans
CD248 Xenopus tropicalis
CD248 Ornithorhynchus anatinus
CD248 Monodelphis domestica
CD248 Homo sapiens
CD248 Mus musculus
Clec14 Carcharodon carcharias
Clec14 Scyliorhinus canicula
Clec14 Latimeria chalumnae
Clec14 Xenopus tropicalis
Clec14 Danio rerio
Clec14 Microcaecilia unicolor
Clec14 Homo sapiens
Clec14 Mus musculus
Clec14 Gallus gallus
Clec14 Corvus moneduloides
Clec14 Trachemys scripta elegans
Clec14 Lacerta agilis
CD93 Microcaecilia unicolor
CD93 Xenopus tropicalis
CD93 Carcharodon carcharias
CD93 Scyliorhinus canicula
CD93 Latimeria chalumnae
CD93 Danio rerio
CD93 Gallus gallus
CD93 Corvus moneduloides
CD93 Trachemys scripta elegans
CD93 Lacerta agilis
CD93 Ornithorhynchus anatinus
CD93 Monodelphis domestica
CD93 Homo sapiens
CD93 Mus musculus

Topology 8

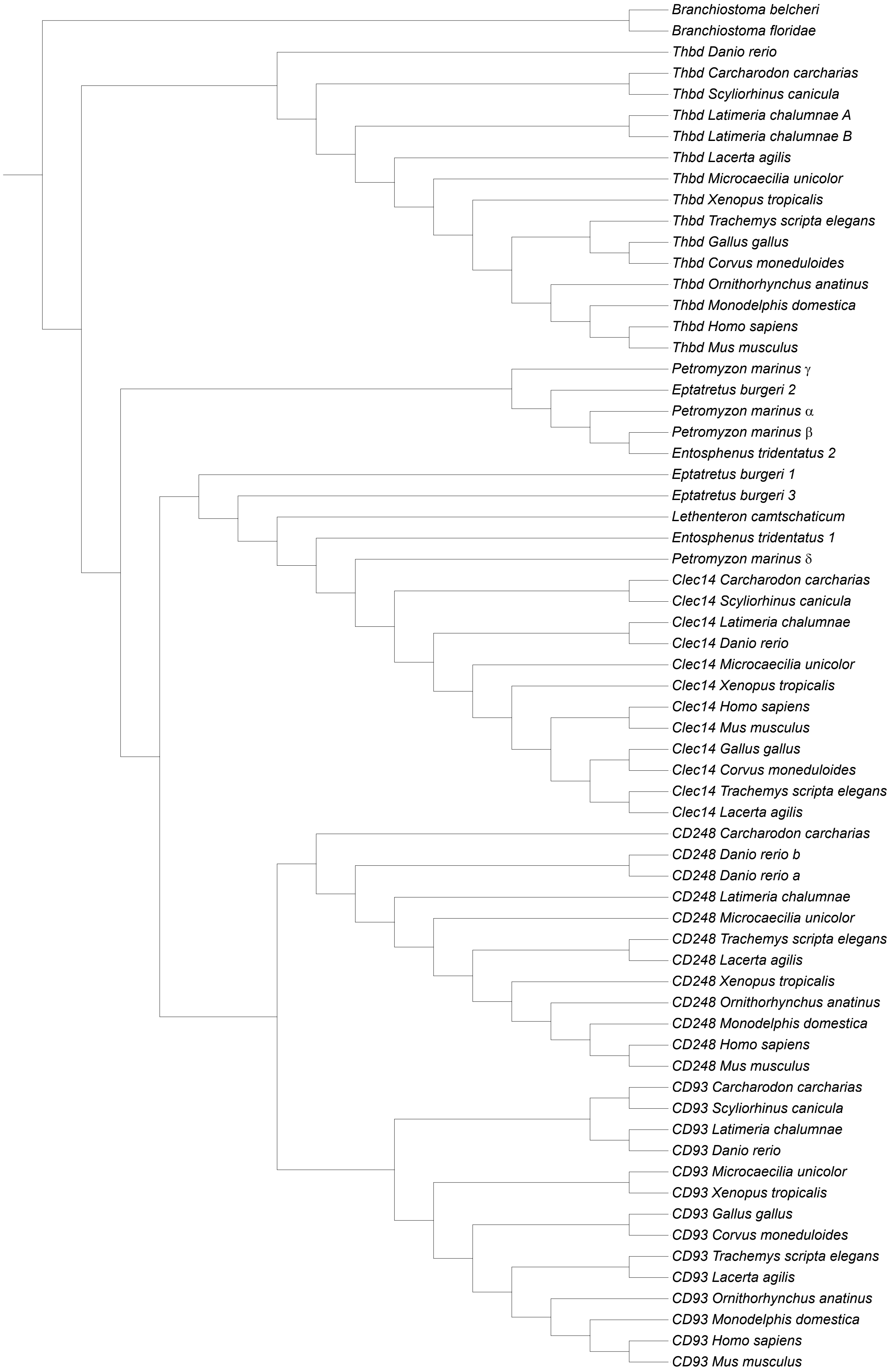
Branchiostoma belcheri
Branchiostoma floridae
Thbd Danio rerio
Thbd Carcharodon carcharias
Thbd Scyliorhinus canicula
Thbd Latimeria chalumnae A
Thbd Latimeria chalumnae B
Thbd Lacerta agilis
Thbd Microcaecilia unicolor
Thbd Xenopus tropicalis
Thbd Trachemys scripta elegans
Thbd Gallus gallus
Thbd Corvus moneduloides
Thbd Ornithorhynchus anatinus
Thbd Monodelphis domestica
Thbd Homo sapiens
Thbd Mus musculus
Petromyzon marinus γ
Eptatretus burgeri 2
Petromyzon marinus α
Petromyzon marinus β
Entosphenus tridentatus 2
Eptatretus burgeri 1
Eptatretus burgeri 3
Lethenteron camtschaticum
Entosphenus tridentatus 1
Petromyzon marinus δ
Clec14 Carcharodon carcharias
Clec14 Scyliorhinus canicula
Clec14 Latimeria chalumnae
Clec14 Danio rerio
Clec14 Microcaecilia unicolor
Clec14 Xenopus tropicalis
Clec14 Homo sapiens
Clec14 Mus musculus
Clec14 Gallus gallus
Clec14 Corvus moneduloides
Clec14 Trachemys scripta elegans
Clec14 Lacerta agilis
CD248 Carcharodon carcharias
CD248 Danio rerio b
CD248 Danio rerio a
CD248 Latimeria chalumnae
CD248 Microcaecilia unicolor
CD248 Trachemys scripta elegans
CD248 Lacerta agilis
CD248 Xenopus tropicalis
CD248 Ornithorhynchus anatinus
CD248 Monodelphis domestica
CD248 Homo sapiens
CD248 Mus musculus
CD93 Carcharodon carcharias
CD93 Scyliorhinus canicula
CD93 Latimeria chalumnae
CD93 Danio rerio
CD93 Microcaecilia unicolor
CD93 Xenopus tropicalis
CD93 Gallus gallus
CD93 Corvus moneduloides
CD93 Trachemys scripta elegans
CD93 Lacerta agilis
CD93 Ornithorhynchus anatinus
CD93 Monodelphis domestica
CD93 Homo sapiens
CD93 Mus musculus

Topology 9

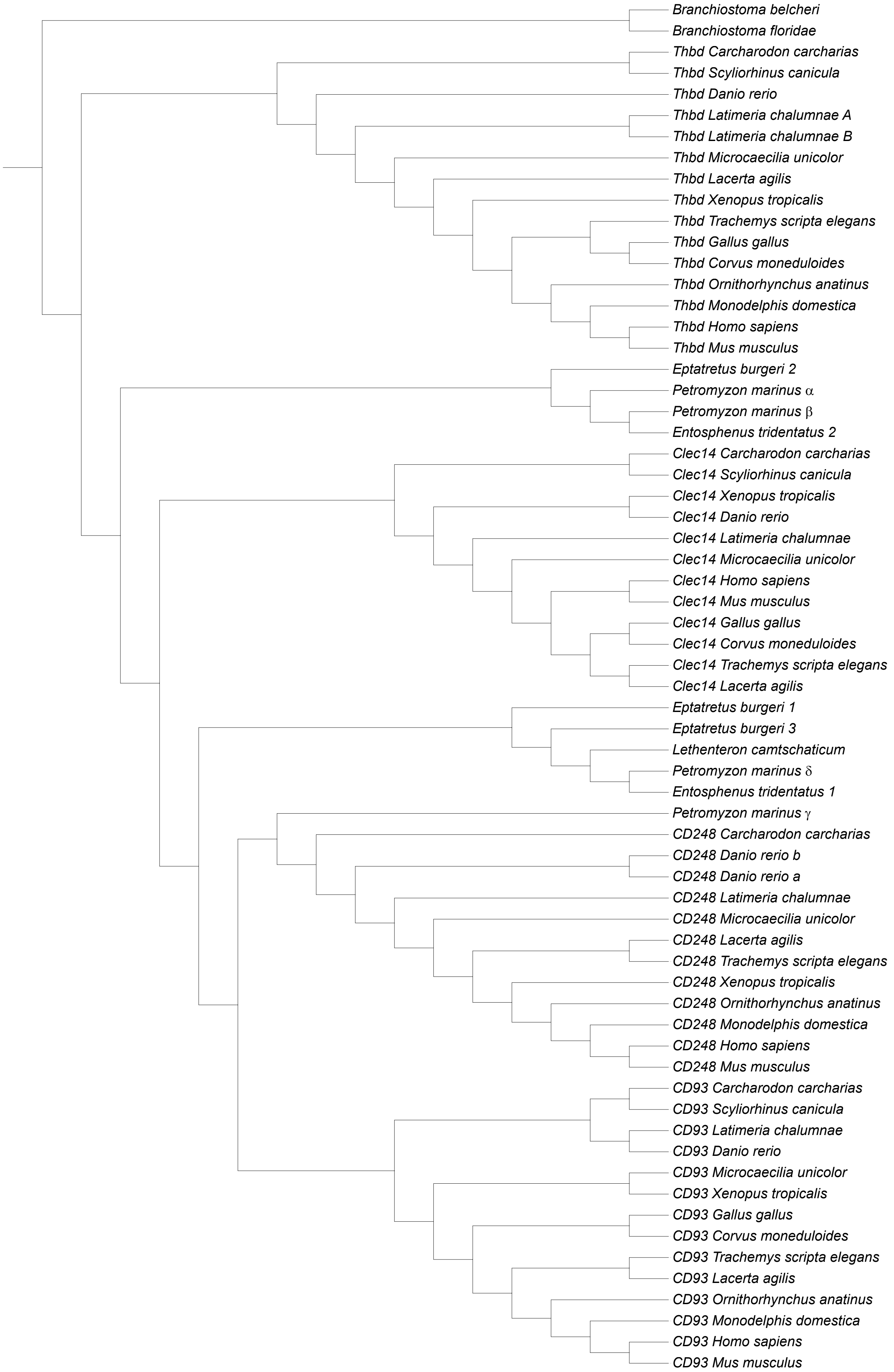
Branchiostoma belcheri
Branchiostoma floridae
Thbd Carcharodon carcharias
Thbd Scyliorhinus canicula
Thbd Danio rerio
Thbd Latimeria chalumnae A
Thbd Latimeria chalumnae B
Thbd Microcaecilia unicolor
Thbd Lacerta agilis
Thbd Xenopus tropicalis
Thbd Trachemys scripta elegans
Thbd Gallus gallus
Thbd Corvus moneduloides
Thbd Ornithorhynchus anatinus
Thbd Monodelphis domestica
Thbd Homo sapiens
Thbd Mus musculus
Eptatretus burgeri 2
Petromyzon marinus α
Petromyzon marinus β
Entosphenus tridentatus 2
Clec14 Carcharodon carcharias
Clec14 Scyliorhinus canicula
Clec14 Xenopus tropicalis
Clec14 Danio rerio
Clec14 Latimeria chalumnae
Clec14 Microcaecilia unicolor
Clec14 Homo sapiens
Clec14 Mus musculus
Clec14 Gallus gallus
Clec14 Corvus moneduloides
Clec14 Trachemys scripta elegans
Clec14 Lacerta agilis
Eptatretus burgeri 1
Eptatretus burgeri 3
Lethenteron camtschaticum
Petromyzon marinus δ
Entosphenus tridentatus 1
Petromyzon marinus γ
CD248 Carcharodon carcharias
CD248 Danio rerio b
CD248 Danio rerio a
CD248 Latimeria chalumnae
CD248 Microcaecilia unicolor
CD248 Lacerta agilis
CD248 Trachemys scripta elegans
CD248 Xenopus tropicalis
CD248 Ornithorhynchus anatinus
CD248 Monodelphis domestica
CD248 Homo sapiens
CD248 Mus musculus
CD93 Carcharodon carcharias
CD93 Scyliorhinus canicula
CD93 Latimeria chalumnae
CD93 Danio rerio
CD93 Microcaecilia unicolor
CD93 Xenopus tropicalis
CD93 Gallus gallus
CD93 Corvus moneduloides
CD93 Trachemys scripta elegans
CD93 Lacerta agilis
CD93 Ornithorhynchus anatinus
CD93 Monodelphis domestica
CD93 Homo sapiens
CD93 Mus musculus

Topology 10
