# Supplementary material for: A glimpse into the past: phylogenesis and protein domain analysis of the group XIV of C-type lectins in vertebrates
Source: BMC Genomics. 2022 Jun 4;23:420. doi: 10.1186/s12864-022-08659-6 (PMC9167495; doi:10.1186/s12864-022-08659-6)
Supplement: Supplementary file 2 — Additional file 2: Supplementary Figure 2. Identification of the first CD93 phospho-tyrosine consensus in each group of vertebrates. CD93 first phospho-tyrosine consensus was retrieved by NetPhos3.1 and plotted using WebLogo2.8.2. The predicted consensus for each group of vertebrates is shown as entropy rendering abundance to emphasize motif’s information content. Number of species used for the analysis: Chondrichthyes n = 3, Teleosts n = 27, Amphibians n = 2, Reptiles n = 2, Birds n = 11, Marsupials n = 3 and Mammals n = 17. [file 12864_2022_8659_MOESM2_ESM.pdf]

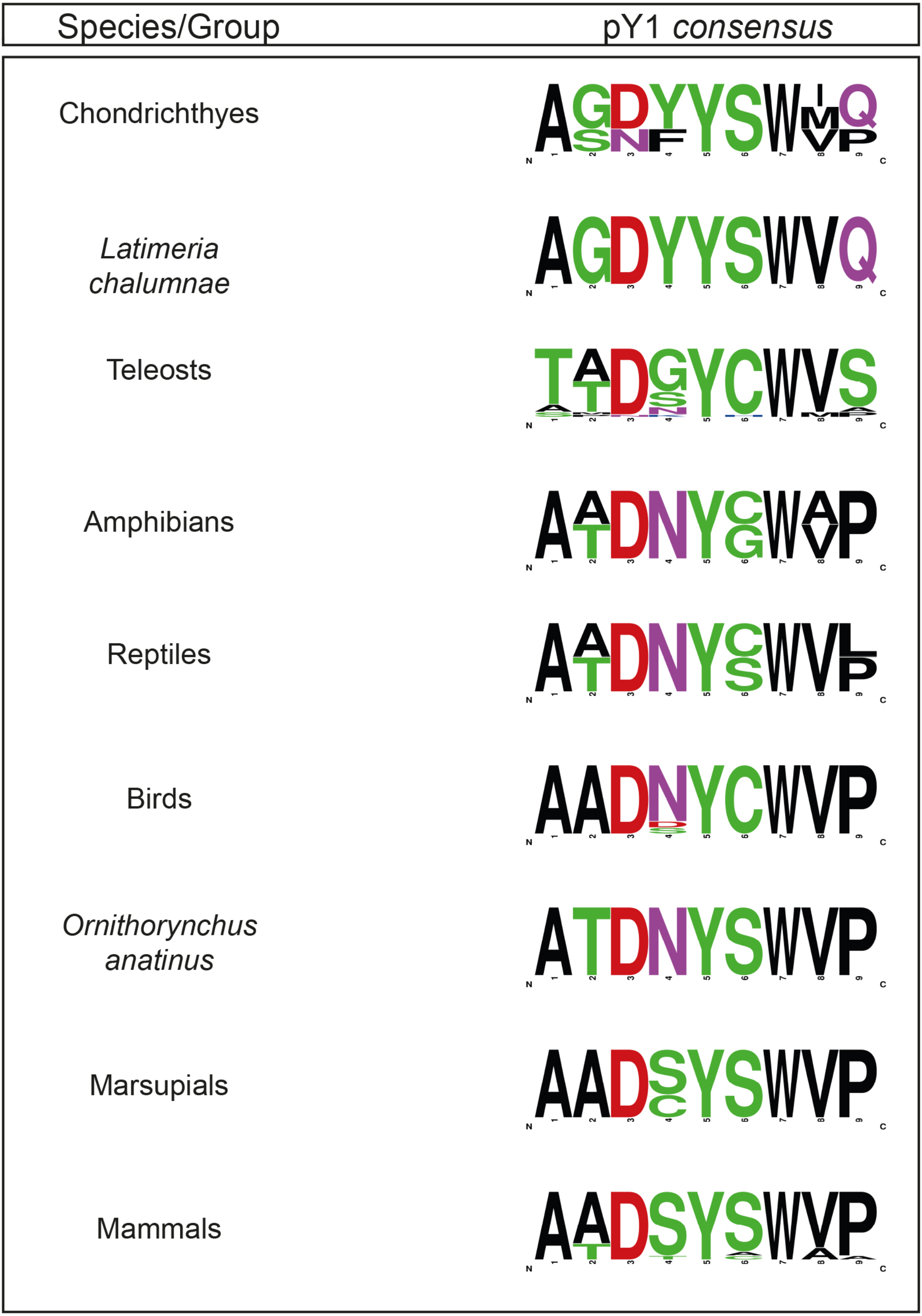
Species/Group
pY1 *consensus*
Chondrichthyes
Latimeria chalumnae
Teleosts
Amphibians
Reptiles
Birds
Ornithorynchus anatinus
Marsupials
Mammals
