# Supplementary figures and images for: A glimpse into the past: phylogenesis and protein domain analysis of the group XIV of C-type lectins in vertebrates
Source: BMC Genomics. 2022 Jun 4;23:420. doi: 10.1186/s12864-022-08659-6 (PMC9167495; doi:10.1186/s12864-022-08659-6)

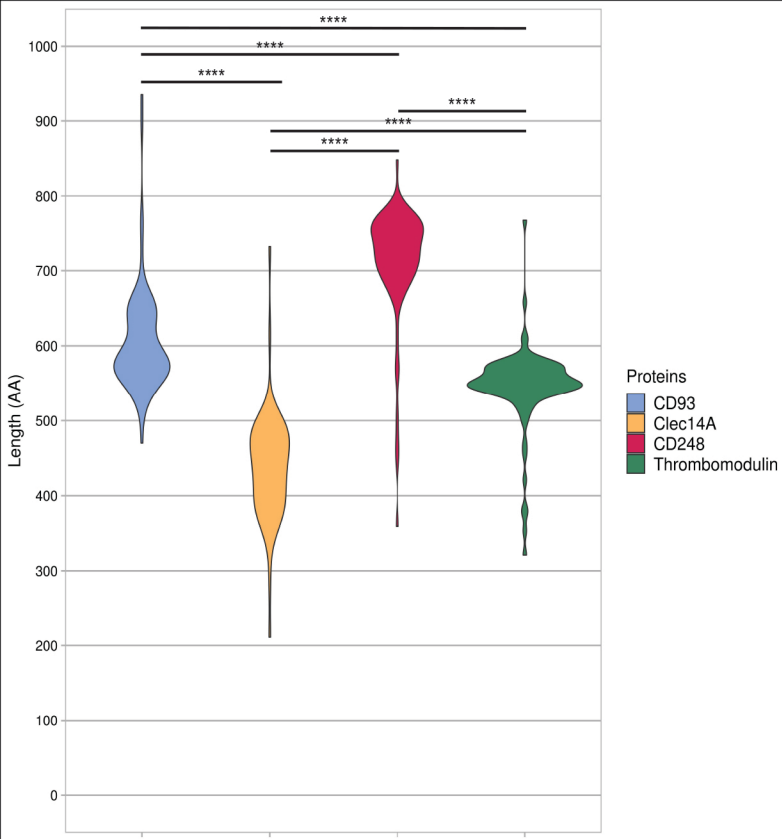

Supplement: Supplementary file 3 — Additional file 3: Supplementary Figure 3. Average protein length of group XIV CTLDcps. Protein length was calculated and plotted in the R environment v.3.6.3. Number of species used for the analysis: CD93 n= 98, Clec14A n= 64, CD248 n= 61 and Thrombomodulin n= 88. Bars show SD. ****P < 0.0001 One-way ANOVA test. [file 12864_2022_8659_MOESM3_ESM.pdf]
